# Materials process informatics-assisted precise particle size control of metal–organic frameworks

**DOI:** 10.1039/d6sc03212e

**Published:** 2026-06-20

**Authors:** Yuan Wang, Heng Liu, Yusuke Hashimoto, Kazuyuki Iwase, Hao Li, Takaaki Tomai

**Affiliations:** a Institute of Multidisciplinary Research for Advanced Materials, Tohoku University 2-1-1 Katahira, Aoba-ku Sendai 980-8577 Japan takaaki.tomai.e6@tohoku.ac.jp; b Graduate School of Engineering, Tohoku University 6-6-11 Aramaki-aza Aoba, Aoba-ku Sendai 980-8579 Japan; c Advanced Institute for Materials Research (WPI-AIMR), Tohoku University Sendai 980-8577 Japan; d Frontier Research Institute for Interdisciplinary Sciences, Tohoku University Sendai 980-8577 Japan

## Abstract

Precise control over the particle size of metal–organic frameworks (MOFs) is pivotal for optimizing their performance in catalysis, separation, and drug delivery. However, conventional synthetic strategies largely depend on empirical trial-and-error, which lacks predictive power and fails to decode the complex interplay between nucleation and growth kinetics. Herein, we report a materials process informatics framework for the predictive control of MOF particle size, using zeolitic imidazolate framework-8 (ZIF-8) as a representative model system. A comprehensive database was constructed through systematic curation from the literature, with seven process descriptors employed as input features. Multiple machine-learning algorithms were benchmarked, among which the Categorical Boosting (CB) model achieved the best predictive performance after hyperparameter optimization, with a coefficient of determination (*R*^2^) of 0.90 on the test set. Furthermore, SHapley Additive exPlanations (SHAP) analysis identified the precursor concentration ratio and reaction time as the most influential parameters governing particle size. Experimental validation using an automated synthesis platform showed excellent agreement between predicted and measured particle sizes, confirming the model's robustness and predictive reliability. Overall, the proposed framework enables intelligent synthesis optimization and targeted experimental design, thereby providing a practical route toward controllable MOF synthesis. This work demonstrates how materials process informatics can shift MOF particle-size engineering from empirical optimization toward data-driven design, offering a broadly applicable strategy for advanced materials synthesis.

## Abstract 2

Interpretable machine learning transforms a literature-derived database into predictive particle-size control for metal–organic frameworks.

## Graphical abstract

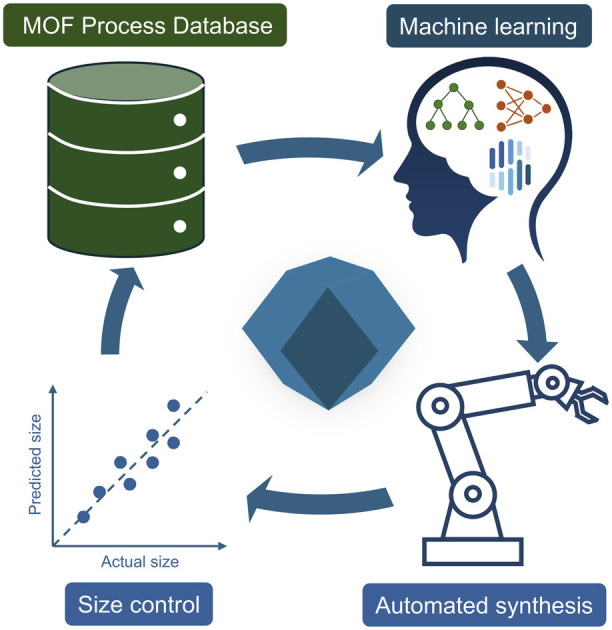

## Introduction

Metal–organic frameworks (MOFs), a class of crystalline porous materials assembled from metal nodes (ions or clusters) and organic linkers, have attracted considerable attention due to their tunable structures and versatile functionalities.^1^ By varying the metal centers and linker motifs, MOFs with diverse topologies and well-defined pore environments can be systematically designed and synthesized.^2,3^ As a result, MOFs have shown considerable promise in a wide range of applications, including gas storage and separation, heterogeneous catalysis, and biomedical fields such as drug delivery and therapy.^4–7^

Zeolitic imidazolate framework-8 (ZIF-8), constructed from zinc ions (Zn^2+^) and 2-methylimidazole (2-HmIm) linkers, has emerged as one of the most representative MOF systems because of its remarkable chemical and thermal stability.^8^ Importantly, the performance of ZIF-8 is governed by complex structure–property relationships, in which particle size serves as a critical parameter. In general, smaller particles are favourable for catalysis and drug delivery, as they can increase surface accessibility and shorten diffusion distances.^9–11^ However, when ZIF-8 is used as a sacrificial template or precursor, excessively small particles may become structurally vulnerable during pyrolysis, thereby compromising the integrity and durability of the derived catalytic materials.^12,13^ In membrane-based separation, particle size also strongly affects interfacial compatibility and defect formation, both of which are closely related to separation performance.^14,15^ Therefore, precise control of ZIF-8 particle size during synthesis is essential for optimizing performance across diverse functional domains.

The synthesis of ZIF-8 is governed by a multidimensional process space defined by precursor concentration, ligand-to-metal ratio, reaction time, temperature, and other synthesis variables.^16–19^ Despite this complexity, current strategies for particle size control remain largely empirical, typically relying on iterative trial-and-error adjustment of synthesis conditions based on literature protocols or prior experience.^20^ Such approaches are not only time-consuming and resource-intensive, but also make it difficult to capture complex parameter interactions and non-linear dependencies that govern particle formation. Consequently, the precise and reproducible synthesis of ZIF-8 with target particle sizes remains a significant challenge.

Materials informatics has emerged as an important paradigm in modern materials science by accelerating the discovery and optimization of functional materials through the integration of data science and domain knowledge. Within this context, machine learning (ML) has become a particularly powerful approach because it enables predictive models to be constructed directly from experimental and computational datasets.^21,22^ In synthesis research, this concept further extends to materials process informatics, where multidimensional process variables are correlated with target material characteristics to guide rational process design.^23,24^ This paradigm has shifted materials research from trial-and-error experiments toward data-driven strategies, particularly in nanomaterial synthesis.^25,26^ Recent studies have demonstrated the potential of machine learning for predicting particle size from synthesis parameters. Liu *et al.* demonstrated that random forest models could accurately predict the phase and classify particle sizes into broad categories such as nano, sub-micron, and micron, while the model failed to predict the exact particle size.^27^ Pellegrino *et al.* developed a stacking ensemble model to estimate the size of ZnO nanoparticles, but the relatively small dataset used for training may limit the robustness of the resulting model.^28^ Zhang *et al.* further applied ML regression to Eu-MOF synthesis, but the sparsity of the dataset (27 samples) restricted the analysis mainly to the dominant variable and hindered the identification of more complex multivariate effects.^29^ In addition, machine learning has also been applied to the synthesis analysis of ZIF-8.^30,31^ Allegretto *et al.* proposed a unified roadmap for ZIF-8 nucleation and growth by analyzing how synthetic variables influence particle size and morphology under water- and methanol-based conditions, providing valuable insights into the synthesis mechanism of ZIF-8.^31^ Despite these advances, the development of more accurate predictive frameworks for experimentally guided particle size control across multidimensional synthesis conditions remains an important direction.

In this study, we develop a materials process informatics framework to predict the particle size of ZIF-8 as a function of synthesis parameters. Multiple regression algorithms are systematically benchmarked to determine the optimal predictive model. Model interpretability is established using SHapley Additive exPlanations (SHAP) to quantify the contribution of each process parameter and identify the key descriptors governing particle size. The predictive capability of the resulting model is further evaluated through independent synthesis experiments conducted by an automatic synthesis system. By integrating predictive modelling with feature-importance analysis, this study achieves exceptional size prediction and provides meaningful insight into the process–size relationship, thereby clarifying how key synthesis parameters govern particle size evolution. Overall, these results demonstrate the potential of machine learning to move MOF synthesis beyond empirical optimization toward more rational and data-guided process design.

## Experimental and methods

### Chemicals and materials

All the reagents and solvents were used without further purification. Zinc nitrate hexahydrate (Zn(NO_3_)_2_·6H_2_O, 99%), 2-methylimidazole (C_4_H_6_N_2_, 98%), and methanol (CH_3_OH) were purchased from FUJIFILM Wako Pure Chemical Corporation.

### Synthesis of ZIF-8 for model validation

To evaluate model accuracy, a series of independent experiments were conducted. Specifically, 2-methylimidazole and Zn(NO_3_)_2_·6H_2_O were separately dissolved in equal volumes of methanol or water under continuous magnetic stirring to prepare solution A and solution B, respectively. Solution A was subsequently added to solution B through an automatic synthesis system to generate a homogeneous mixture. After a certain reaction time, the resulting suspension was centrifuged at 10 000 rpm for 4 minutes to collect the solid product. The obtained precipitate was washed 3 times with methanol to remove unreacted precursors and then dried at 60 °C for 12 hours to yield the final ZIF-8 powder. The detailed process parameters for these experiments are summarized in Table 1. To enhance experimental precision and minimize deviation by human error, an automated synthesis system was employed. The detailed equipment specifications and photographs of the system are provided in Fig. S1. The particle size of these samples was measured using ImageJ software.

**Table 1:** Summary of experimental conditions for model validation

Sample	*C*_Zn_	*C*_2-HmIm_/*C*_Zn_	Solvent amount (mL)	Reaction time (min)	Stirring	Solvent	Temperature (°C)	Predicted size (nm)	Actual size (nm)
1	0.40	8	20	1440	T	Methanol	25	365	290
2	0.13	4	60	1440	T	Methanol	25	236	210
3	0.40	16	20	30	T	Methanol	25	184	204
4	0.13	8	60	120	F	Methanol	25	130	141
5	0.08	40	100	60	T	Water	25	344	292
6	0.20	16	140	720	Initial	Methanol	25	252	228
7	0.05	38	100	1440	T	Water	25	1372	1382
8	0.08	16	100	30	T	Methanol	25	78	61

### Characterization

The crystallization properties of ZIF-8 were characterized by X-ray diffraction (XRD) using a Rigaku SmartLab 9MTP diffractometer equipped with a Cu Kα radiation source (*λ* = 1.5406 Å). The morphologies and particle sizes of the synthesized ZIF-8 were measured using a scanning electron microscope (SEM, JSM-7800F).

### Database creation

The dataset used in this study was established through a systematic literature survey of the Web of Science database using the keyword “ZIF-8 size”. An initial search returned 2392 records, of which 2304 remained after excluding review articles. Titles and abstracts were then manually screened to identify studies explicitly reporting the synthesis of ZIF-8. To ensure data consistency, only studies employing Zn(NO_3_)_2_ and 2-methylimidazole (2-HmIm) as precursors were retained. The target variable was average particle size, which was determined from scanning electron microscopy (SEM) or transmission electron microscopy (TEM) images.

Seven synthesis parameters were used as input features, including the zinc precursor concentration (*C*_Zn_), ligand-to-metal concentration ratio (*C*_2-HmIm_/*C*_Zn_), solvent type (water or methanol), solvent volume, reaction time, reaction temperature, and stirring conditions (continuous stirring, denoted as T; stirring only during the initial mixing step, denoted as Initial; and quiescent synthesis, denoted as F). These parameters were selected based on the fundamental mechanism of particle formation, which involves nucleation and crystal growth. In particular, *C*_Zn_ and *C*_2-HmIm_/*C*_Zn_ govern the degree of supersaturation and therefore strongly influence nucleation behaviour and the formation of primary nuclei. Solvent type also plays an important role because its physicochemical properties, such as dielectric constant, dipole moment, and van der Waals volume, affect solubility and surface energy, thereby modulating nucleation behavior.^32^ During the subsequent growth stage, particle size is further influenced by supersaturation, which determines the supply of building units and the rate of diffusion-limited growth, whereas reaction time governs the extent of growth and possible coarsening associated with Ostwald ripening.^33^ Temperature exerts a coupled effect on both thermodynamic and kinetic factors, including solubility, interfacial energy, diffusion, and attachment rate, thereby exerting a strong influence on particle size.^34^ In addition, solvent volume and stirring conditions were included because they influence mixing uniformity and mass transport, both of which are important for achieving homogeneous crystal growth.^35^

### ML algorithms and evaluation methods

A comprehensive benchmarking study was performed to evaluate the predictive performance of seven representative machine learning (ML) algorithms. The models included Random Forest (RF), eXtreme Gradient Boosting (XGB), Categorical Boosting (CB), Light Gradient Boosting Machine (LGBM), Support Vector Regression (SVR), K-Nearest Neighbors (KNN), and Multilayer Perceptron (MLP), covering ensemble approaches, kernel-based methods, instance-based learning, and neural networks. The XGB and CB models were implemented using the xgboost and catboost libraries,^35,36^ respectively, whereas the remaining algorithms were developed with the scikit-learn package (version 1.4.2) in Python.^37^

Before model training, the database was randomly divided into training and testing subsets using an 80 : 20 split ratio with a fixed random state of 60. Continuous variables were standardized to have a mean of 0 and a standard deviation of 1, thereby eliminating scale disparities among descriptors. Except for the CB model, categorical variables were converted into numerical features using one-hot encoding to ensure compatibility with machine learning algorithms. Hyperparameter optimization was conducted by a randomized search of the parameter space with 100 iterations combined with 5-fold cross-validation (CV).^38^ Model performance was evaluated using three standard regression metrics: the coefficient of determination (*R*^2^), root-mean-square error (RMSE), and mean absolute error (MAE).^39^

## Results and discussion

### Workflow

**Fig. 1:**
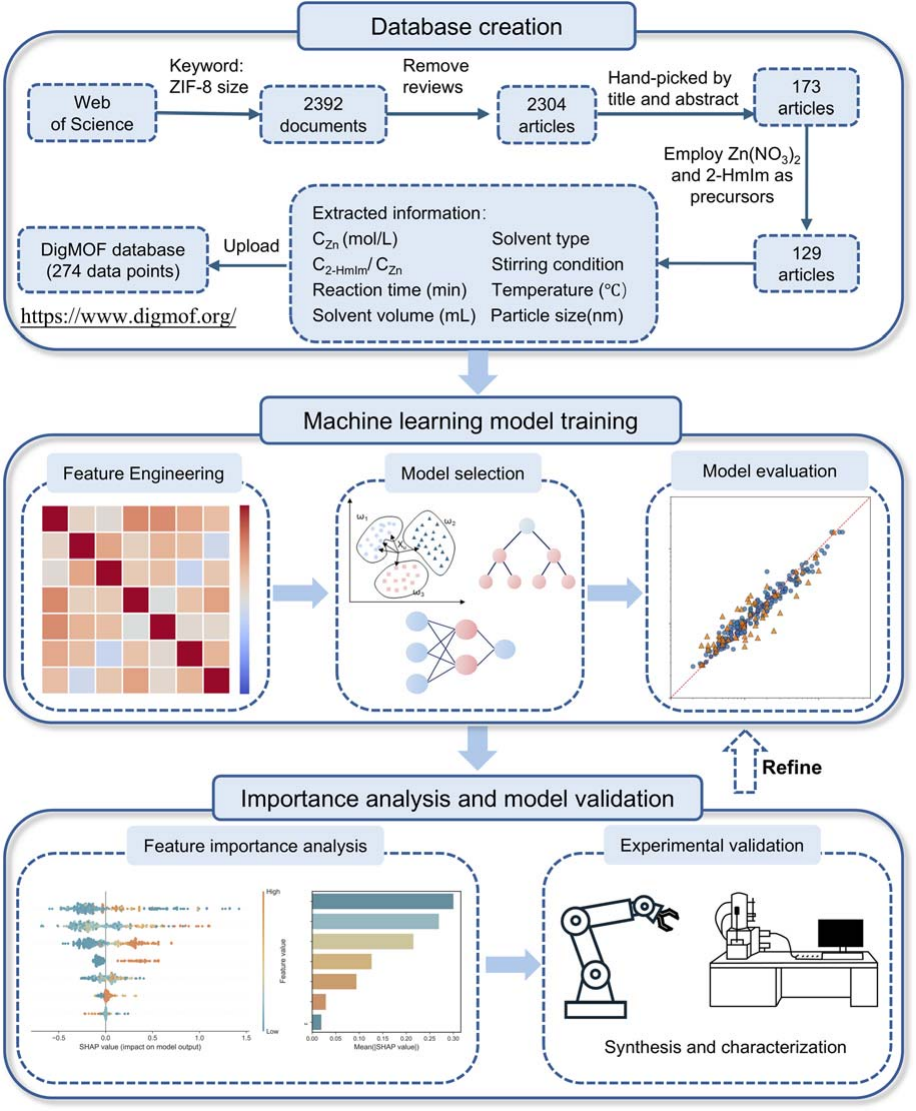
Proposed workflow of materials process informatics-assisted size control of MOFs.

Fig. 1 illustrates the overall workflow of the materials process informatics framework for size control of ZIF-8 particles. A literature-curated database was first established through systematic screening and data extraction, from which key synthesis parameters were defined as input descriptors, and particle size was used as the target. The resulting dataset was subsequently subjected to feature engineering, model training, and performance evaluation to identify an appropriate regression model for particle-size prediction. Feature-importance analysis was then performed to quantify the relative contributions of individual synthesis parameters. Feature importance analysis was then performed to reveal the relative contributions of each synthesis parameter. Finally, the model was experimentally validated through an automated synthesis platform, and the newly generated data provide a basis for iterative refinement of the predictive framework.

### Database creation

After extraction, 274 valid data points were obtained from 130 articles covering 17 years from 2009 to 2025. The literature sources included in the database are summarized in Table S1. As shown in Fig. S2, these sources form an interconnected citation network, reflecting the cumulative development of ZIF-8 synthesis research, in which many later studies were derived from or built upon previously reported protocols. The color scale further shows that these publications span multiple years, reflecting the temporal distribution of the literature considered in this work. In addition, a digital MOF platform (DigMOF, https://www.digmof.org/) was developed to integrate the curated dataset, enabling dynamic data visualization, precise literature tracking, and efficient data management. The corresponding user interface is shown in Fig. S3.

**Fig. 2:**
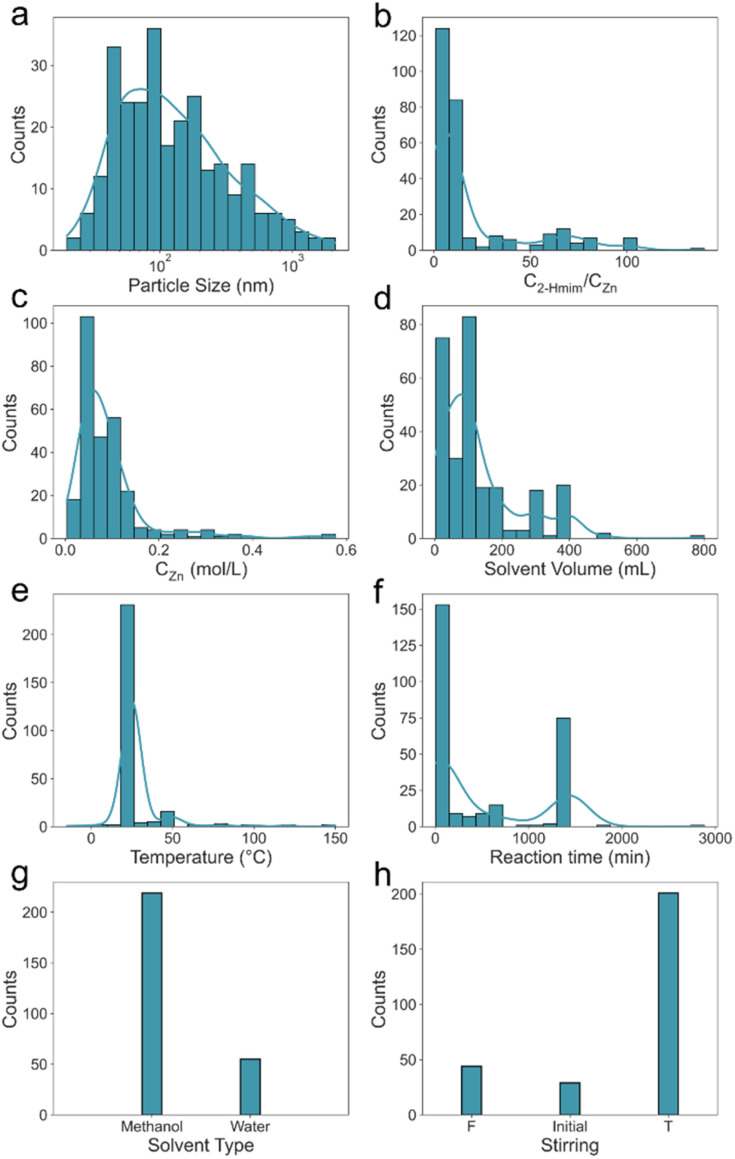
Data distributions of synthesis parameters and particle size in the database: (a) particle size, (b) *C*_2-HmIm_/*C*_Zn_, (c) *C*_Zn_, (d) solvent amount, (e) temperature, (f) reaction time, (g) solvent type, and (h) stirring conditions.

Fig. 2 presents the distributions of particle size and the seven synthesis descriptors. The dataset covers a relatively broad synthesis domain, with particle size spanning a wide range, indicating substantial variability in the reported ZIF-8 crystals under different preparation conditions. Pronounced right-skewed distributions are observed for *C*_Zn_, *C*_2-HmIm_/*C*_Zn_, and solvent volume, suggesting that most reported syntheses were conducted within relatively limited parameter ranges. Reaction temperature is strongly centred around 25 °C, reflecting the predominance of room-temperature synthesis in the literature. In contrast, reaction time exhibits a bimodal distribution, with one cluster at short durations on the order of tens of minutes and another extending to long durations exceeding 1000 min. For the categorical descriptors, methanol is used more frequently than water, and continuous stirring is much more common than either initial stirring only or quiescent synthesis. Collectively, these results indicate that the dataset spans a diverse but unevenly sampled synthesis space, reflecting the intrinsic heterogeneity of literature-derived experimental data. To prepare the dataset for machine learning, all continuous variables were standardized to remove differences in scale, whereas categorical variables were converted into numerical features through one-hot encoding.

**Fig. 3:**
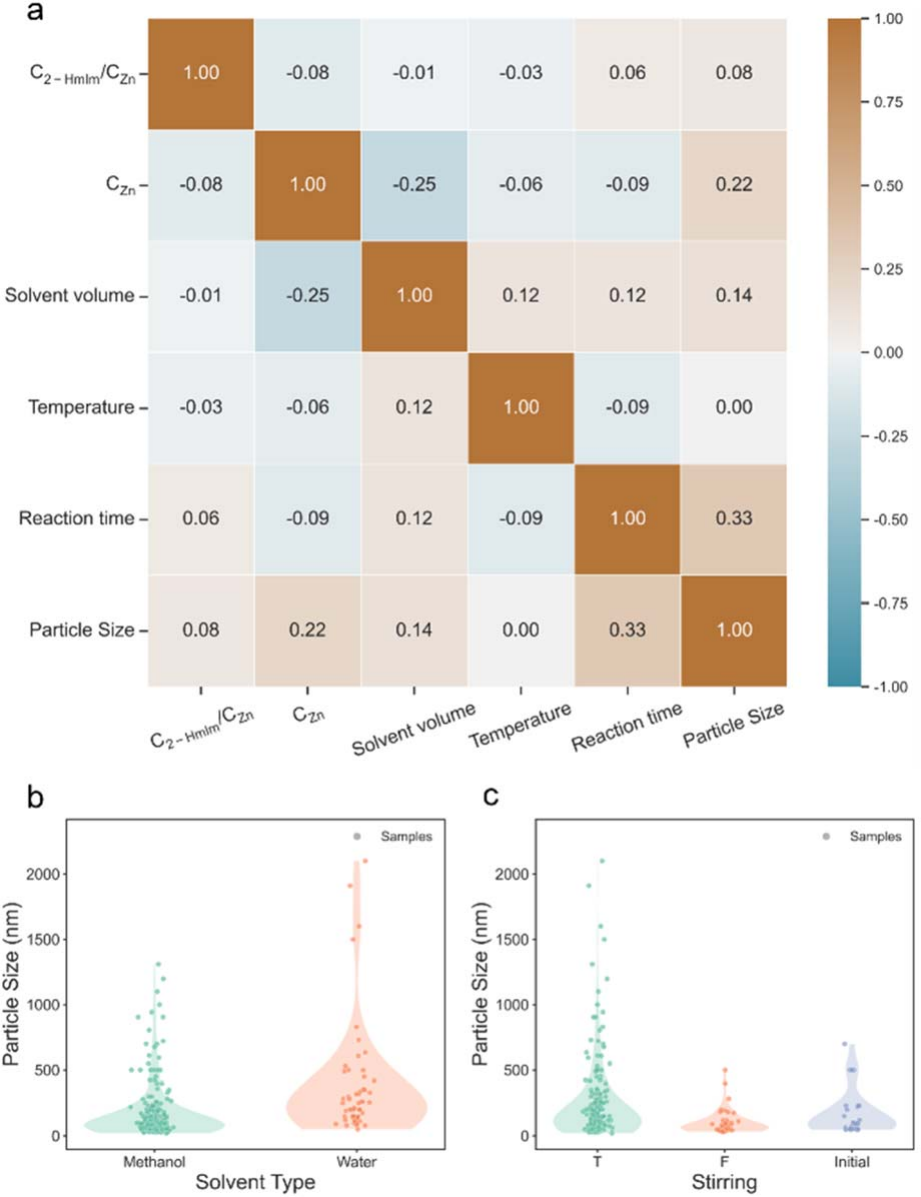
(a) Spearman correlation coefficients between pairs of numerical synthesis parameters and between each parameter and the target particle size; (b and c) violin plot showing the influence of solvent type and stirring conditions on particle size.

To further elucidate the relationships among numerical process parameters and their association with particle size, a Spearman correlation matrix was constructed (Fig. 3a). Strong correlations between input variables (|*ρ*| > 0.8) are generally undesirable because they may introduce redundancy and reduce model robustness. In this database, most descriptors show only weak pairwise correlations (|*ρ*| < 0.3), indicating minimal collinearity and a high degree of statistical independence among the input variables. With respect to particle size, reaction time shows the highest positive correlation, followed by *C*_Zn_ and solvent volume, whereas temperature exhibits almost no linear correlation with particle size. These weak correlations indicate that particle size cannot be explained by any single descriptor alone, highlighting the complexity of the underlying synthesis–size relationship. The categorical effects are further visualized in Fig. 3b and c. Water-based syntheses tend to yield larger particles with a broader size distribution than methanol-based systems. In addition, continuously stirred systems show a wider spread in particle size than quiescent or initially stirred conditions. Overall, these results highlight the importance of machine learning for capturing the coupled effects of multiple synthesis parameters on particle size.

### Model selection and evaluation

**Fig. 4:**
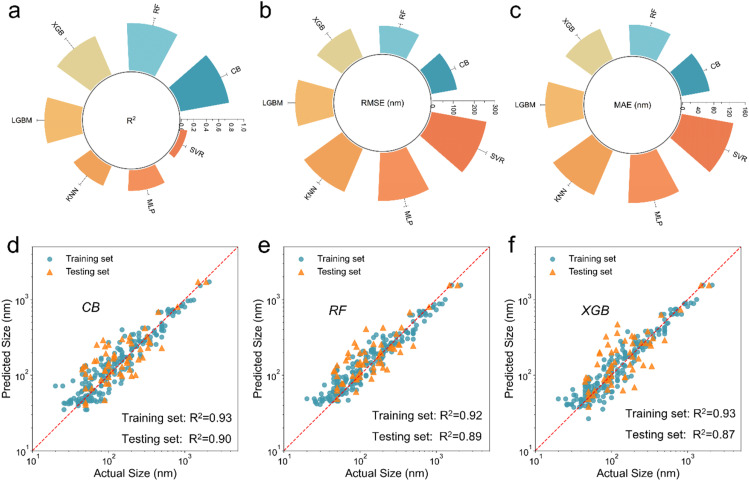
Performance comparison of machine learning models evaluated by 5-fold cross-validation in terms of (a) *R*^2^, (b) RMSE, and (c) MAE, with error bars indicating the standard deviation across the five folds. The performance of the hyperparameter-optimized models on the training and hold-out test sets is further presented for (d) CB, (e) RF, and (f) XGB models.

Seven supervised learning algorithms with default hyperparameters were first benchmarked to identify suitable models for ZIF-8 particle-size prediction. The ensemble tree-based models were included because of their ability to capture nonlinear feature interactions and their strong robustness for relatively small datasets.^36,40–42^ SVR and KNN were included as representatives of kernel-based and instance-based learning paradigms, respectively, while MLP was selected as a prototypical neural network model capable of learning complex nonlinear mappings.^43–45^ As shown in Fig. 4a–c, clear differences in predictive performance were observed among the tested models. Overall, the ensemble tree-based algorithms exhibited the strongest performance. Among them, the CB model achieved the best overall accuracy, with the highest *R*^2^ value (0.79) and the lowest RMSE and MAE. RF and XGB also showed strong predictive capability, whereas KNN, MLP, and SVR performed substantially worse. Collectively, these results identify ensemble learning methods, particularly CB, RF, and XGB, as the most suitable candidates for subsequent hyperparameter optimization and feature-importance analysis.

Hyperparameter optimization was performed using a randomized search strategy within predefined parameter spaces, combined with 5-fold cross-validation to ensure robust model training and reliable parameter selection. The optimal hyperparameters obtained for each model are summarized in Table S2. Following optimization, the final models were retrained on the full training dataset and evaluated on an independent hold-out test set to assess their predictive performance. As shown in Fig. 4d–f, all three ensemble models demonstrated strong predictive performance, with predicted particle sizes closely matching the experimental values. Among these, the CB model delivered the highest accuracy, achieving *R*^2^ values of 0.93 for the training set and 0.90 for the testing set, thereby establishing CB as the most accurate and reliable algorithm for predicting ZIF-8 particle size.

### Importance analysis

**Fig. 5:**
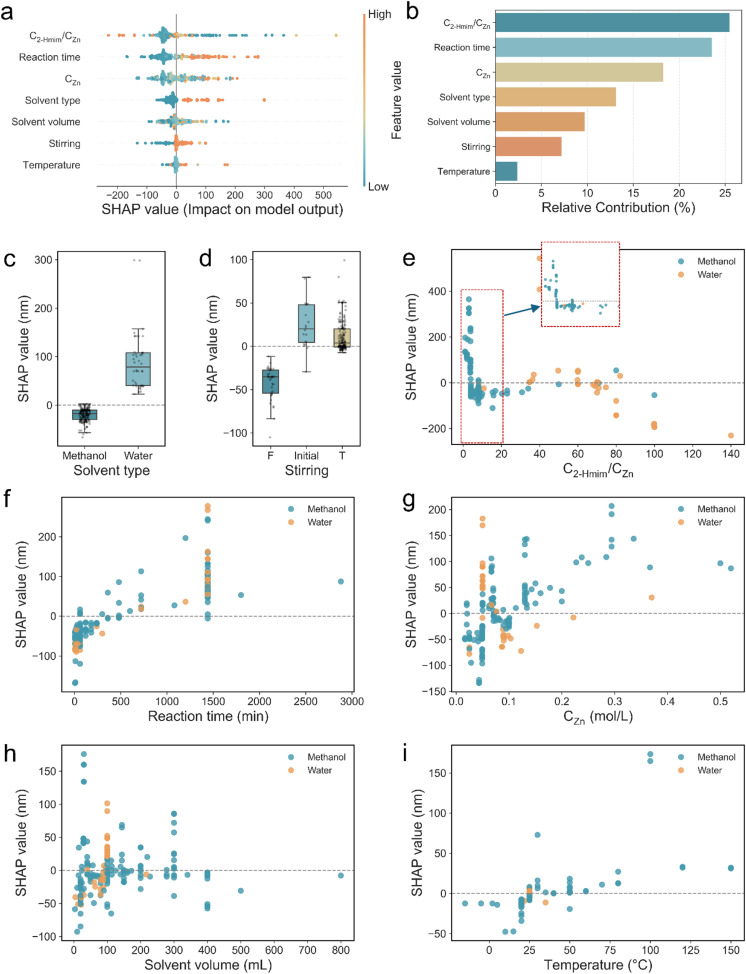
SHAP analysis of the CB model, including (a) the global SHAP summary plot, (b) the SHAP-based feature importance ranking of all features, and SHAP dependence plots for (c) solvent type, (d) stirring conditions, (e) *C*_2-HmIm_/*C*_Zn_, (f) reaction time, (g) *C*_Zn_, (h) solvent volume, and (i) temperature. Points are colored by solvent type (blue: methanol; orange: water). The inset in (c) provides a magnified view of the low-ratio region (0–20).

To interpret the optimized models and identify the dominant synthesis parameters governing particle size, SHapley Additive exPlanations (SHAP) analysis was performed. SHAP quantifies the contribution of each input descriptor to the model output and further reveals whether a given feature value drives the prediction toward larger or smaller particle sizes. Firstly, the SHAP summary plot presents the overall distribution of feature contributions across the dataset, with each point corresponding to an individual sample. The accompanying bar chart provides a quantitative ranking of feature importance based on the relative contributions derived from normalized mean absolute SHAP values. As shown in Fig. 5a, b and S4, the three optimized ensemble models exhibit highly consistent importance rankings, indicating that the identified descriptor effects are robust across different models. In all cases, *C*_2-HmIm_/*C*_Zn_ emerged as the most influential parameter, exhibiting the highest relative contribution of around 25%. Reaction time was identified as the second most important factor, followed by *C*_Zn_. Solvent volume and solvent type have moderate contributions, whereas stirring conditions and temperature contribute much less to the final prediction. The relatively low SHAP importance of temperature is likely attributed to the narrow temperature range represented in the literature dataset, with most syntheses being conducted near room temperature.

To further elucidate the effects of individual synthesis parameters, SHAP dependence plots derived from the optimized CB model were analyzed. SHAP values quantify how each feature value shifts the prediction relative to the baseline, defined as the average model output. Positive SHAP values indicate a tendency toward larger predicted particle sizes, whereas negative values correspond to smaller predicted sizes. As shown in Fig. 5c, the solvent type exhibits a pronounced and systematic effect on the predicted particle size. Methanol is predominantly associated with negative SHAP values, indicating a tendency toward smaller particles, whereas water yields mainly positive SHAP values, corresponding to larger predicted sizes. This difference is consistent with the distinct physicochemical properties of the two solvents. The lower polarity of methanol can reduce precursor solubility and promote higher supersaturation, thereby favouring rapid nucleation and suppressing subsequent crystal growth.^46^ In contrast, the higher polarity and stronger coordination ability of water can stabilize Zn^2+^ species in solution, moderate supersaturation, and shift the system toward more growth-dominated regimes.^47^ On this basis, the effects of the main synthesis descriptors were further interpreted with explicit consideration of solvent type.

In comparison, stirring conditions show a much weaker effect on the model output (Fig. 5d). Although slight differences are observed among the three stirring modes, their interpretation remains limited because the literature data generally do not provide sufficiently detailed information on stirring speed or intensity. A more comprehensive assessment of stirring effects would require controlled experiments with well-defined mixing parameters, which will be examined in future work.

Among the continuous descriptors, the precursor-related parameters are the most influential. As shown in Fig. 5e, the distribution of *C*_2-HmIm_/*C*_Zn_ is strongly separated by solvent type, with methanol-based samples concentrated mainly in the low-ratio region and water-based samples extending to much higher values. The inset highlights the low-ratio range that contains most methanol-derived data points. Despite this disparity, both solvents display a similar overall trend, in which increasing *C*_2-HmIm_/*C*_Zn_ shifts the SHAP value toward more negative contributions, indicating smaller predicted particle sizes. This observation is consistent with classical nucleation–growth theory, wherein low linker-to-metal ratios suppress nucleation and facilitate the formation of larger crystals, whereas high ratios generate abundant nuclei that compete for available precursors and ultimately yield smaller particles.^48,49^

Reaction time also exhibits an overall positive dependence (Fig. 5f), with longer synthesis durations corresponding to more positive SHAP values. This trend is consistent with Ostwald ripening, wherein smaller crystallites dissolve and redeposit onto larger ones, leading to progressive coarsening until thermodynamic equilibrium is reached.^50^

A generally positive trend is observed for *C*_Zn_ (Fig. 5g). At concentrations below 0.1 mol L^−1^, the SHAP values fluctuate around zero, likely because particle size in this regime is strongly influenced by other synthesis variables. As *C*_Zn_ increases, the SHAP values become increasingly positive, indicating a tendency toward larger predicted particle sizes. High precursor concentrations dominantly accelerate primary nucleation and crystal growth through increased supersaturation. Moreover, the resulting high particle density enhances inter-particle collisional growth, and the rapid coalescence of primary clusters and secondary nuclei also leads to the formation of larger particles.^51^

Solvent volume (Fig. 5h) exhibits a nonmonotonic and relatively dispersed effect on the SHAP values. Most data points are distributed around zero, suggesting that the influence of solvent volume is not systematic but likely coupled with other synthesis parameters. The large-volume systems, where extended diffusion distances allow local concentration gradients to persist, induce spatial inhomogeneity, creating microenvironments that promote uneven nucleation and localized aggregation. Therefore, the solvent volume mainly affects not particle size but particle size distribution.

Reaction temperature (Fig. 5i) does not exhibit a clear trend in SHAP values, indicating the absence of systematic temperature dependence within the present dataset. Most data points are concentrated in the near-ambient temperature range and show SHAP values close to zero, although a few higher-temperature samples display positive deviations. This is likely due to the narrow temperature range reported in the literature, as most syntheses were conducted under near-ambient conditions.

Overall, SHAP analysis reveals a physically meaningful interpretation of the factors controlling ZIF-8 particle size. In detail, precursor-related descriptors, particularly *C*_2-HmIm_/*C*_Zn_ and *C*_Zn_, together with reaction time, make the dominant contributions to the model output, whereas solvent-related variables provide additional modulation. Notably, the SHAP dependence plots of the RF and XGB models shown in Fig. S5 and S6 exhibit similar trends for the major descriptors, indicating that the identified parameter effects are consistent across different models. This mechanistic insight enables more rational synthesis design, in which target particle sizes can be approached through systematic adjustment of the key process variables identified by the feature-importance analysis.

### Experimental validation

To further evaluate the predictive reliability of the developed framework, eight independent validation experiments were carried out under synthesis conditions that were not included in the original database. Given that tree-based algorithms such as the CB model generally have limited extrapolation capability outside the descriptor domain represented in the database, the validation experiments were intentionally designed within the experimentally accessible synthesis parameter space covered by the literature-derived dataset. Meanwhile, the reaction temperature was fixed at 25 °C to focus on the effects of the more influential synthesis variables while minimizing uncertainty associated with the sparsely represented temperature conditions in the dataset. To further clarify the relationship between the validation samples and the original database, principal component analysis (PCA) was performed. As shown in Fig. S7, all eight validation samples are located within the main descriptor space covered by the literature-derived dataset, rather than in regions far outside the distribution of the dataset, indicating that the validation experiments mainly assess the interpolative reliability of the model within the investigated synthesis space. Although the validation is mainly interpolative, the curated dataset spans a relatively broad range of synthesis conditions, allowing the model reliability to be evaluated across a broad synthesis space. The detailed experimental conditions are summarized in Table 1.

**Fig. 6:**
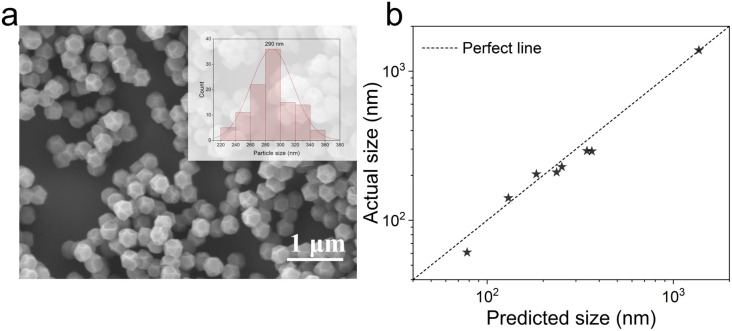
Experimental validation of the predictive model. (a) SEM image of a representative ZIF-8 sample, with the corresponding particle-size distribution shown in the inset, obtained from statistical analysis of more than 100 particles. (b) Comparison between the predicted and experimentally measured particle sizes; the dashed line represents perfect agreement.

Fig. S8 shows the XRD patterns of the prepared samples. All the diffraction peaks of the samples are consistent with the standard PDF card of ZIF-8 (JCPDS 00-062-1030), indicating the successful synthesis of ZIF-8 and the well-preserved framework structure. The SEM images and corresponding particle-size distributions of all samples are provided in Fig. S9. A representative SEM image is shown in Fig. 6a, where the obtained ZIF-8 particles exhibit a well-defined polyhedral morphology. For statistical reliability, particle sizes were determined from more than 100 individually measured particles for each sample, and the average value was taken as the experimental particle size. As shown in Fig. 6b, the predicted particle sizes agree closely with the experimentally measured values over a broad size range.

The observed particle size differences among the validation samples are also consistent with the SHAP analysis of the dominant synthesis descriptors and provide practical guidelines for directional particle size control. For synthesizing larger ZIF-8 particles, conditions with positive SHAP contributions should be selected, particularly water-based synthesis environments, longer reaction times, and suitable *C*_2-HmIm_/*C*_Zn_ values. For example, sample 7 exhibited the largest particle size because of the combined positive SHAP contributions of its key synthesis descriptors. As shown in Fig. 3, the water-based synthesis environment generally contributes more positively to particle growth than methanol-based systems. In addition, the long reaction time of 1440 min corresponds to a strongly positive SHAP region, indicating prolonged crystal growth after nucleation. The *C*_2-HmIm_/*C*_Zn_ value of 38 is also located within a positive SHAP region under the water-based environment. These factors collectively promote the formation of large ZIF-8 particles. In contrast, smaller particles are favoured by methanol-based synthesis and short reaction time, as observed for sample 8. In addition, sample 3 exhibited a larger particle size than sample 8, which can be mainly associated with its higher *C*_Zn_. A higher *C*_Zn_ may increase the concentration of primary nuclei and enhance the probability of inter-particle collision, aggregation, and secondary particle formation, ultimately contributing to larger particle sizes. Therefore, reducing *C*_Zn_, together with using methanol-based synthesis and limiting prolonged growth, can be an effective strategy for obtaining smaller ZIF-8 particles within the investigated synthesis space. These results further confirm the robustness of the proposed framework and demonstrate its capability to reliably guide the synthesis of ZIF-8 with targeted particle sizes under diverse reaction conditions.

## Conclusions

In conclusion, this work establishes a materials process informatics framework for the predictive control of ZIF-8 particle size. The optimized CB model achieved the best performance, with an *R*^2^ of 0.90. Interpretable SHAP analysis further identified the precursor concentration ratio and reaction time as the dominant parameters governing particle size. These findings provide a holistic insight into the particle formation mechanism by clarifying how process parameters affect nucleation and crystal growth. Experimental validation under synthesis conditions outside the database confirmed the framework's predictive accuracy and robustness. However, the model remains limited in resolving temperature-dependent effects because of the narrow temperature variation represented in the current dataset. In addition, incomplete reporting of stirring speed and mixing intensity in the literature limits quantitative assessment of hydrodynamic influences on particle size distribution. Future work will focus on expanding the accessible synthesis space through automated and systematically designed experiments, while incorporating underrepresented process variables to improve model generalizability and predictive resolution. Overall, the framework developed here is readily transferable to other MOFs and related porous materials and highlights the broader potential of data-driven approaches to move synthesis research from empirical optimization toward predictive design.

## Author contributions

Yuan Wang: writing – original draft, writing – review & editing, methodology, investigation, conceptualization. Heng Liu: writing – review & editing, methodology. Yusuke Hashimoto: writing – review & editing, methodology. Kazuyuki Iwase: writing – review & editing. Hao Li: supervision, writing – review & editing. Takaaki Tomai: supervision, writing – review & editing, resources.

## Conflicts of interest

The authors declare that they have no known competing financial interests or personal relationships that could have appeared to influence the work reported in this paper.

## Supplementary Material

SC-OLF-D6SC03212E-s001

## Data Availability

The database supporting this article has been uploaded to a digital MOF platform (DigMOF, https://www.digmof.org/). The curated dataset and source code supporting this article are available at https://github.com/digmof/MOF_size_prediction. Supplementary information (SI) is available. See DOI: https://doi.org/10.1039/d6sc03212e.
