# Supplementary material for: Materials process informatics-assisted precise particle size control of metal–organic frameworks
Source: Chem Sci. 2026 Jun 20;17(31):15034–43. doi: 10.1039/d6sc03212e (PMC13312054; doi:10.1039/d6sc03212e)
Supplement: Supplementary file 1 [file SC-017-D6SC03212E-s001.pdf]

Supplementary Information (SI) for Chemical Science.
This journal is © The Royal Society of Chemistry 2026

Supporting Information

Materials process informatics-assisted precise particle size control of metal-organic frameworks

Yuan Wang,[ab] Heng Liu,[c] Yusuke Hashimoto,[d] Kazuyuki Iwase,[a] Hao Li *[c] and Takaaki Tomai *[ad]

[a] Institute of Multidisciplinary Research for Advanced Materials, Tohoku University, 2-1-1 Katahira, Aoba-ku, Sendai, 980-8577, Japan

[b] Graduate School of Engineering, Tohoku University, 6-6-11 Aramaki-aza Aoba, Aoba-ku, Sendai, 980-8579, Japan

[c] Advanced Institute for Materials Research (WPI-AIMR), Tohoku University, Sendai, 980-8577, Japan

[d] Frontier Research Institute for Interdisciplinary Sciences, Tohoku University, Sendai, 980-8577, Japan

*Corresponding author

Details of the automated synthesis system

Experimental validations were conducted using a custom-developed automated synthesis system. The platform integrates two robotic arms, two electronic pipettes, and an electronic balance, all of which are centrally controlled via Python-based programming to enable fully automated operation. Precise control over the dispensing speed and volume of the electronic pipettes, together with highly reproducible positional control provided by the robotic arms, ensures consistent reagent handling and minimizes human-induced variability. As a result, the automated system significantly improves experimental reproducibility and throughput, while enabling efficient and reliable execution of large sets of synthesis experiments.

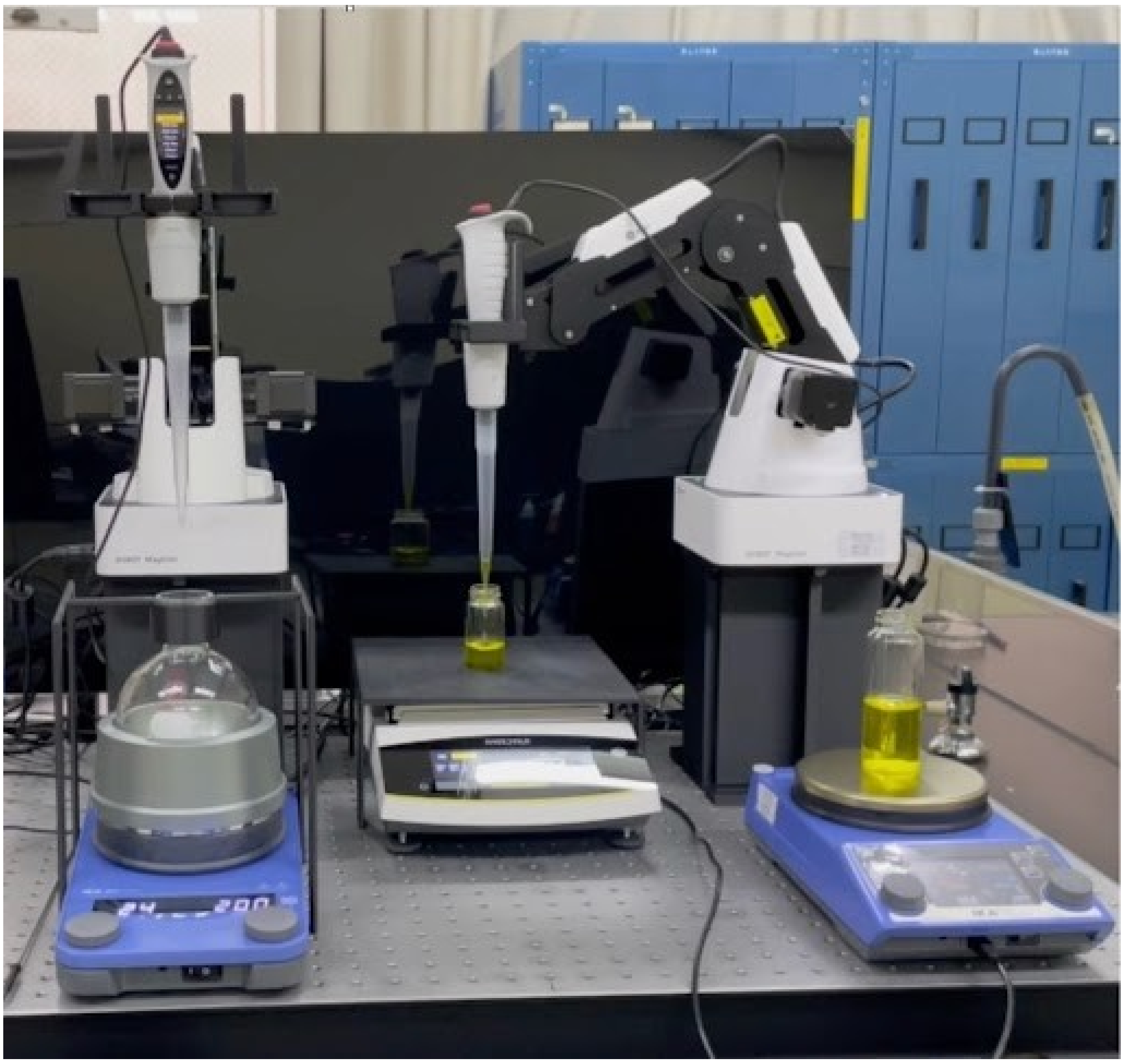

Fig. S1 Photograph of the custom-developed automated synthesis platform used for experimental validation.

Table S1. References included in the curated database.

Author	DOI	Ref
Ahmad (2018)	10.1016/j.seppur.2018.06.067	1
Ahmadi (2024)	10.5812/ijpr-144928	2
Aljundi (2017)	10.1016/j.desal.2017.06.020	3
Armel (2015)	10.3390/catal5031333	4
Balkanloo (2024)	10.1016/j.cej.2024.153835	5
Barjasteh (2022)	10.1016/j.ijpharm.2022.122339	6
Beh (2018)	10.1016/j.matchemphys.2018.06.022	7
Cao (2023)	10.1016/j.desal.2023.116373	8
Chang (2010)	10.1021/ja1058229	9
Chen (2022)	10.1016/j.micromeso.2022.111983	10
Chi (2015)	10.1016/j.memsci.2015.08.016	11
Chin (2018)	10.1039/c8ra03459a	12
Cho (2018)	10.1039/c8ta02797h	13
Chowdhuri (2017)	10.1088/1361-6528/aa57af	14
Cravillon (2009)	10.1021/cm900166h	15
Cravillon (2011a)	10.1002/anie.201102071	16
Cravillon (2011b)	10.1021/cm103571y	17
De (2025)	10.1021/acs.molpharmaceut.4c00981	18
Demir (2014)	10.1016/j.micromeso.2014.07.052	19
Ding (2017)	10.1016/j.colsurfa.2017.02.012	20
Eum (2015)	10.1021/jacs.5b00803	21
Fan (2015)	10.1039/c5ra09981a	22
Gao (2019)	10.1016/j.desal.2017.06.021	23
He (2019)	10.1021/acs.inorgchem.9b00288	24
Heinz (2023)	10.1039/d3qi01007d	25
Imae (2022)	10.1021/acsanm.2c03793	26
Jampa (2020)	10.1016/j.eti.2020.100927	27
Jana (2024)	10.1016/j.colsuc.2024.100030	28
Jian (2015)	10.1039/c5ra04033g	29
Jiang (2013)	10.1021/am403079n	30
Jiang (2017)	10.1021/acsami.7b04432	31
Jiang (2021)	10.1021/acsami.0c22910	32
Jomekian (2016)	10.1016/j.jngse.2016.03.067	33
Jomekian (2017)	10.1016/j.memsci.2016.11.065	34
Karimi (2019)	10.1016/j.seppur.2019.115838	35
Kawada (2022)	10.1039/d2ce00488g	36
Khay (2015)	10.1039/c5ra02636a	37
Kida (2013)	10.1039/c2ce26847g	38

Author	DOI	Ref
Kim (2019)	10.1016/j.memsci.2019.04.029	39
Kim (2024)	10.3390/nano14030284	40
Kolmykov (2016)	10.1016/j.tetlet.2016.11.070	41
Kong (2024)	10.1016/j.surfcoat.2024.130812	42
Kulkarni (2021)	10.1016/j.ijbiomac.2021.02.161	43
Kumari (2013)	10.1021/jp407792a	44
Lai (2014)	10.1080/02726351.2014.920445	45
Lai (2016)	10.1002/adfm.201603607	46
Lai (2017)	10.1021/acscatal.6b02966	47
Lee (2015)	10.1021/acs.jpcc.5b01519	48
Lee (2019a)	10.1016/j.jiec.2018.12.039	49
Lee (2019b)	10.1016/j.memsci.2018.10.015	50
Li (2014)	10.1021/jp508381m	51
Li (2017a)	10.1002/slct.201701607	52
Li (2017b)	10.1039/c6nr08987a	53
Li (2018)	10.1039/c8nr02288g	54
Li (2019)	10.1038/s41467-019-10218-9	55
Li (2020)	10.1246/bcsj.20190298	56
Li (2021a)	10.1016/j.memsci.2021.119095	57
Li (2021b)	10.1016/j.seppur.2021.118794	58
Li (2022)	10.3390/membranes12020122	59
Li (2024)	10.1002/smll.202406187	60
Linder-Patton (2018)	10.1039/c8ce00746b	61
Liu (2013a)	10.1039/c3cc45308a	62
Liu (2013b)	10.1039/c3ta12433a	63
Liu (2014)	10.1016/j.memsci.2013.09.029	64
Liu (2017)	10.1039/c6dt04582k	65
Luo (2018)	10.1002/adma.201704576	66
Luo (2019)	10.1002/cctc.201900051	67
McEwen (2013)	10.1016/j.chemphys.2012.12.012	68
Mittal (2022)	10.1038/s41598-022-14630-y	69
Mohammadi (2024)	10.1021/acsami.3c15524	70
Muñoz-Gil (2019)	10.3390/nano9101369	71
Nguyen (2023)	10.1016/j.jcou.2023.102451	72
Nie (2017)	10.1149/2.1521713jes	73
Pan (2011)	10.1039/c0cc05002d	74
Pandey (2024)	10.1016/j.molstruc.2024.138452	75
Patterson (2015)	10.1021/jacs.5b00817	76
Qin (2023)	10.1016/j.ijpharm.2023.123167	77

Author	DOI	Ref
Qiu (2022)	10.1002/slct.202203273	78
Saghir (2021)	10.1016/j.materresbull.2021.111372	79
Schejn (2014)	10.1039/c3ce42485e	80
Schneider (2024)	10.1002/celc.202300476	81
Seo (2025a)	10.1007/s11814-024-00215-0	82
Seo (2025b)	10.1016/j.jallcom.2025.178578	83
Sharma (2020)	10.1021/acs.jpcc.0c07194	84
Shen (2023)	10.1039/d3ce00162h	85
Shi (2016)	10.1039/c6tb00104a	86
Si (2020)	10.1007/s10853-020-04909-8	87
Song (2023)	10.1021/acsami.3c02647	88
Sánchez-Laínez (2016)	10.1016/j.memsci.2016.05.039	89
Ta (2018)	10.1002/cjce.23155	90
Tanaka (2012)	10.1246/cl.2012.1337	91
Tanaka (2015)	10.1021/acs.jpcc.5b09520	92
Thomas (2025)	10.1039/d5na00217f	93
Tian (2016)	10.1039/c6dt00565a	94
Torad (2013)	10.1039/c3cc38955c	95
Tran (2020)	10.1016/j.seppur.2019.116026	96
Tsai (2016)	10.1016/j.micromeso.2015.08.041	97
Van Cleuvenbergen (2016)	10.1021/acs.chemmater.6b01087	98
Venna (2010)	10.1021/ja109268m	99
Wang (2016)	10.1039/c6ta02420c	100
Wang (2017)	10.1039/c7cy01725a	101
Wang (2018a)	10.1002/adfm.201802596	102
Wang (2018b)	10.1002/smll.201704282	103
Wang (2019)	10.1016/j.chemosphere.2019.06.008	104
Wang (2023)	10.1016/j.apsusc.2022.156181	105
Weber (2020)	10.1021/acs.cgd.9b01444	106
Xue (2024)	10.1007/s10904-024-03253-7	107
Yahia (2021)	10.1016/j.micromeso.2020.110761	108
Yang (2017)	10.1021/acsami.7b10856	109
Yang (2021)	10.1016/j.eurpolymj.2020.110212	110
Yin (2015)	10.1016/j.cej.2014.08.075	111
Zhang (2012)	10.1021/jz300855a	112
Zhang (2013)	10.1021/jz402019d	113
Zhang (2014a)	10.1021/ja5084128	114
Zhang (2014b)	10.1021/jp5081466	115
Zhang (2014c)	10.1039/c4nr00348a	116

Author	DOI	Ref
Zhang (2017)	10.1021/acsami.7b05142	[117]
Zhang (2018)	10.1007/s10853-018-2049-2	[118]
Zhang (2019a)	10.1016/j.micromeso.2018.12.035	[119]
Zhang (2019b)	10.1016/j.micromeso.2019.109568	[120]
Zhang (2025)	10.1021/acsapm.5c00612	[121]
Zhao (2015)	10.1246/cl.150137	[122]
Zheng (2016)	10.1021/jacs.5b11720	[123]
Zhou (2015)	10.1039/c5ta00524h	[124]
Zhou (2023)	10.1002/elan.202200158	[125]
Zhu (2011)	10.1021/cm201701f	[126]
Zhu (2013)	10.1016/j.catcom.2012.12.003	[127]
Zhu (2017)	10.1038/NMAT4852	[128]
Zhu (2018)	10.1021/acsami.8b00072	[129]
Zhu (2024)	10.1016/j.seppur.2023.126209	[130]

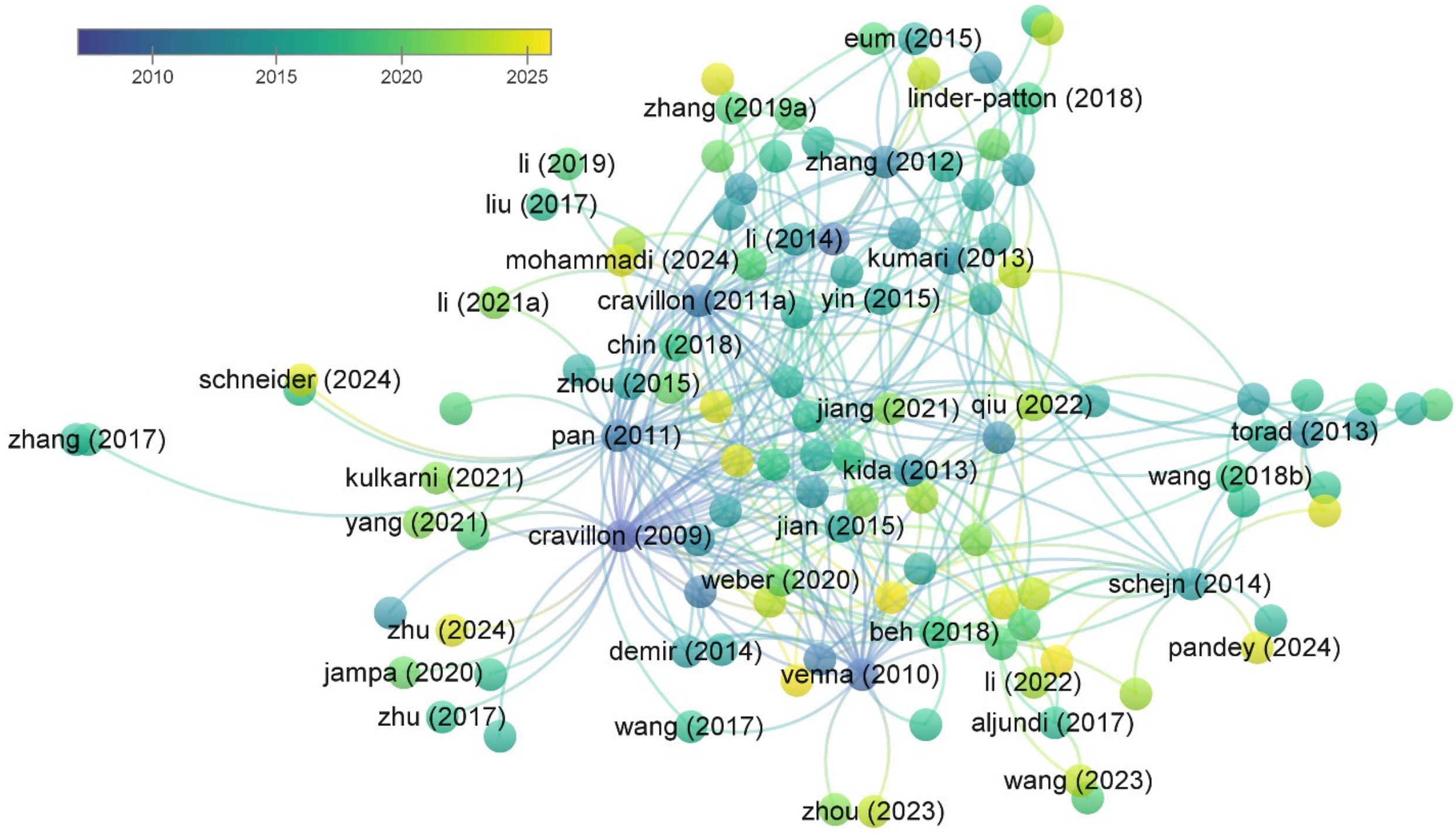

Fig. S2 Citation network of the literature sources included in the dataset. The connecting lines indicate citation relationships between publications, and the node color represents publication year. The network visualization was generated using VOSviewer[131].

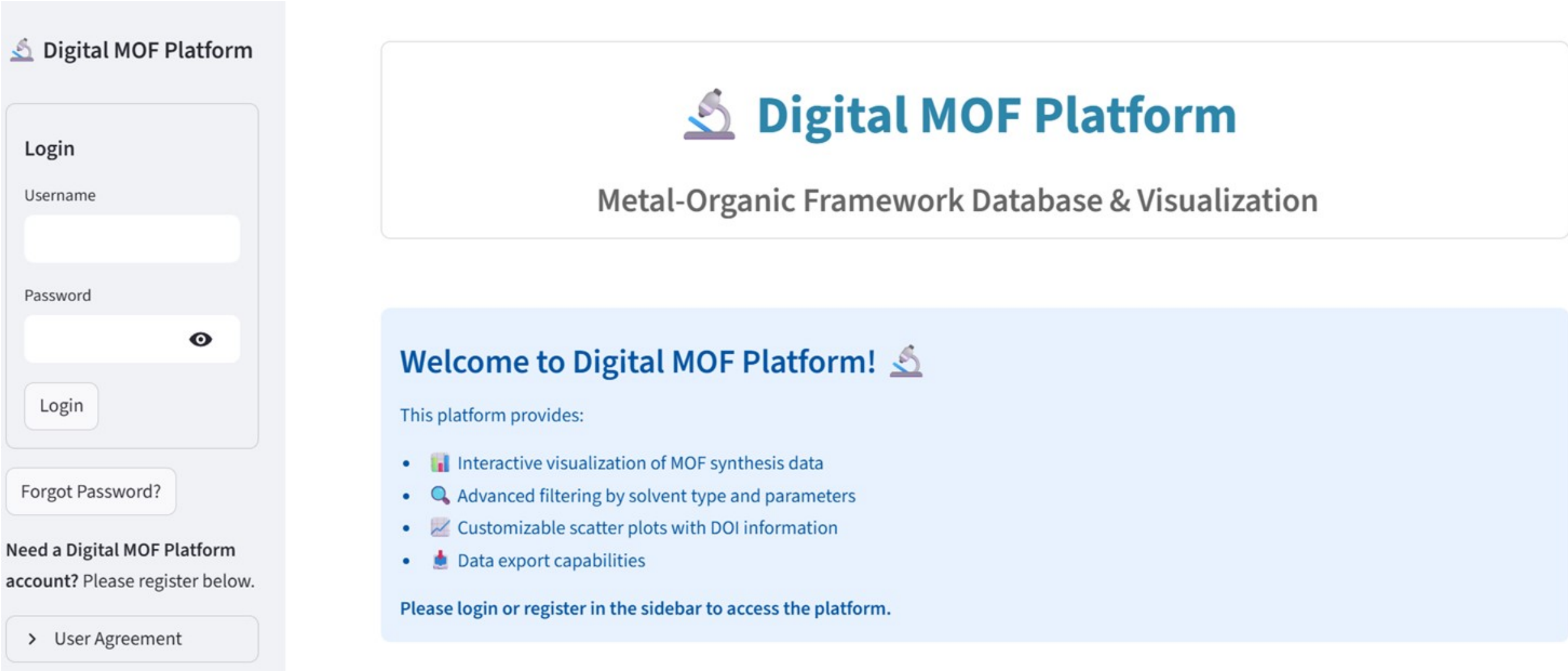

Fig. S3 Interface of the Digital MOF Platform (DigMOF, https://www.digmof.org/) for MOF synthesis data management and visualization.

Table S2. The optimal hyperparameters for CB, RF, and XGB models

**CB**	**RF**	**XGB**
iterations = 200	n_estimators = 150	n_estimators = 150
learning_rate = 0.04	max_depth = 12	colsample_bytree = 0.8
depth = 8	min_samples_split = 4	max_depth = 5
l2_leaf_reg = 1.0	min_samples_leaf = 1	min_child_weight = 2
bagging_temperature=0	max_features = 'log2'	reg_alpha = 5
loss_function = 'RMSE'	ccp_alpha=0.0022	reg_lambda = 5
random_seed = 42	random_state = 42	gamma = 0.1
verbose = False	n_jobs = -1	subsample = 0.8
		learning_rate = 0.07
		random_state = 42
		n_jobs = -1

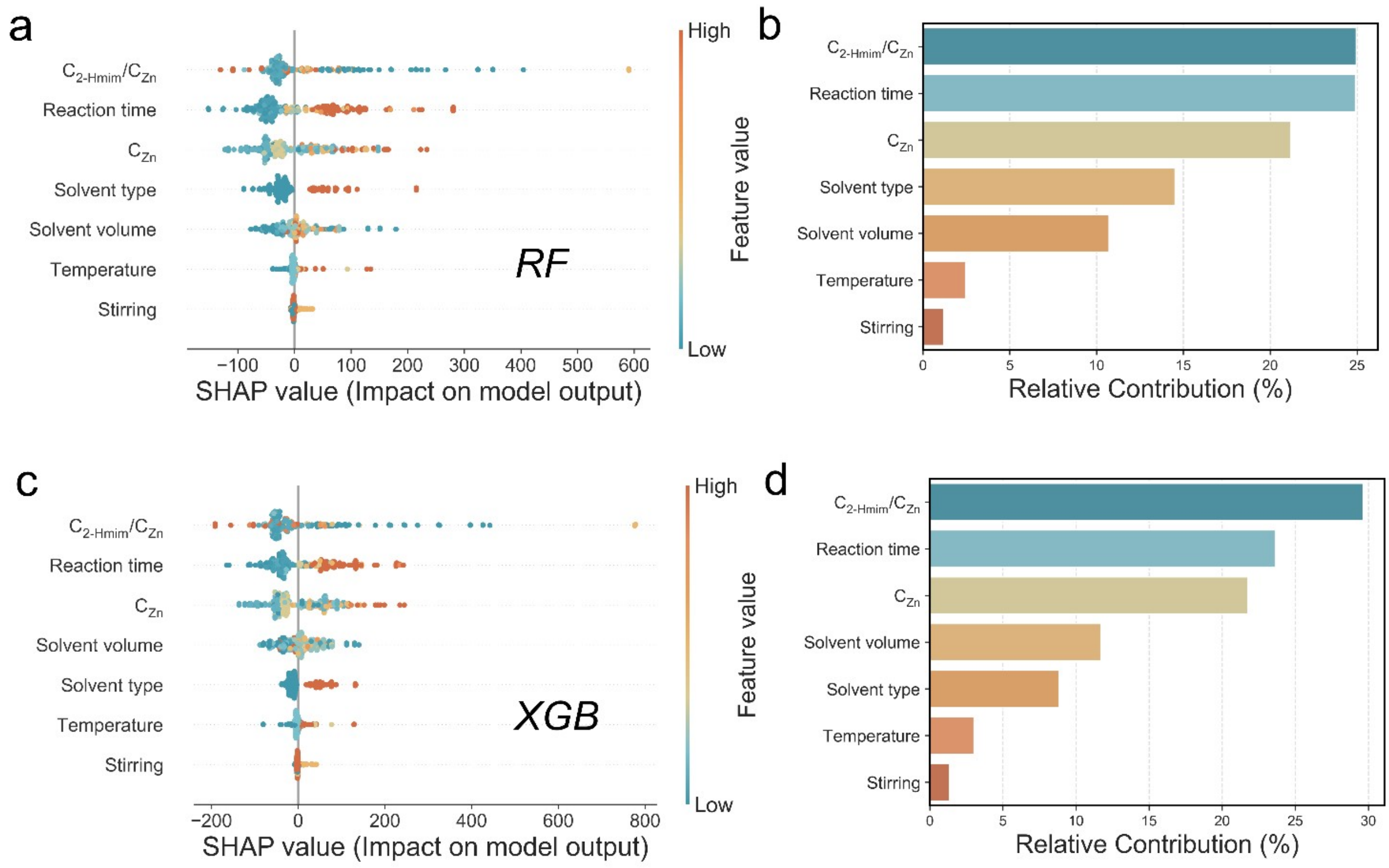

Fig. S4 SHAP-based feature importance analysis. (a, c) SHAP summary plots showing the distribution of the impact of each feature on the model output for RF and XGB models, respectively. (b, d) Corresponding bar charts representing the relative contribution of synthesis parameters.

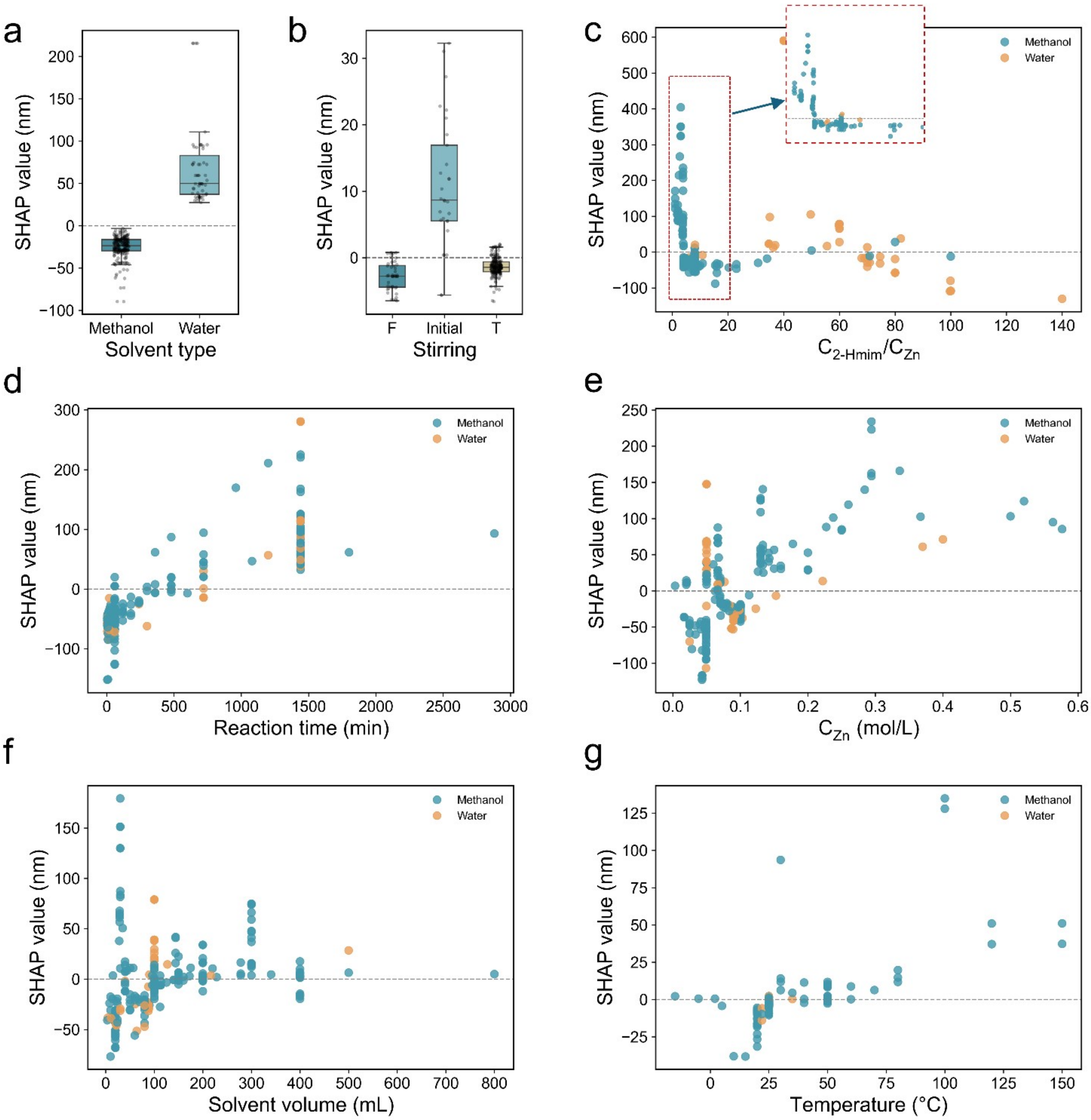

Fig. S5 SHAP dependence analysis of RF model. (a) solvent type, (b) stirring conditions, (c) $C_{2\text{-HmIm}}/C_{Zn}$, (d) reaction time, (e) C_{Zn}, (f) solvent volume, and (g) temperature. Points are colored by solvent type (blue: methanol; orange: water). The inset in (c) provides a magnified view of the low-ratio region (0–20).

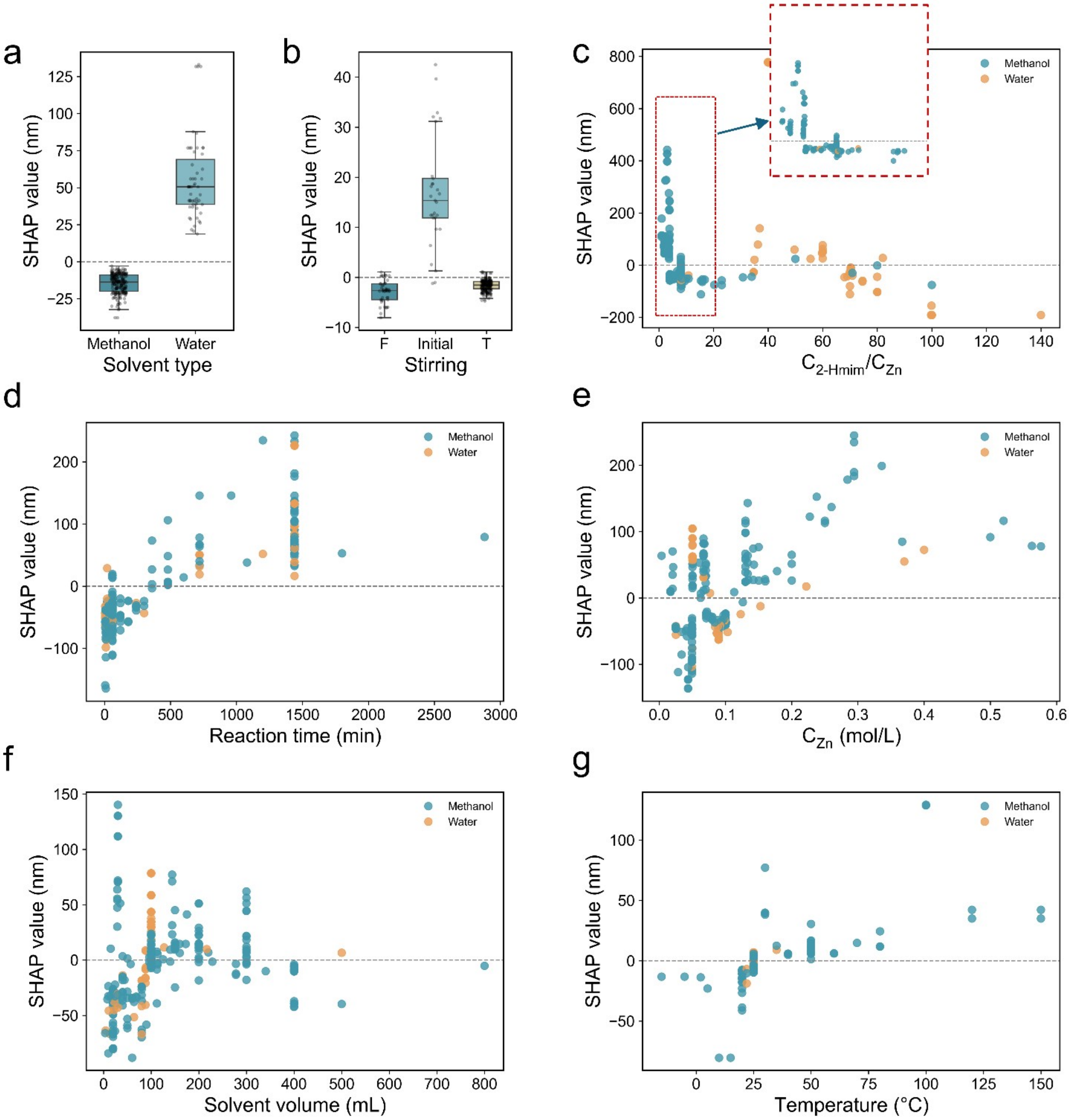

Fig. S6 SHAP dependence analysis of XGB model. (a) solvent type, (b) stirring conditions, (c) $C_{2\text{-HmIm}}/C_{Zn}$, (d) reaction time, (e) C_{Zn}, (f) solvent volume, and (g) temperature. Points are colored by solvent type (blue: methanol; orange: water). The inset in (c) provides a magnified view of the low-ratio region (0–20).

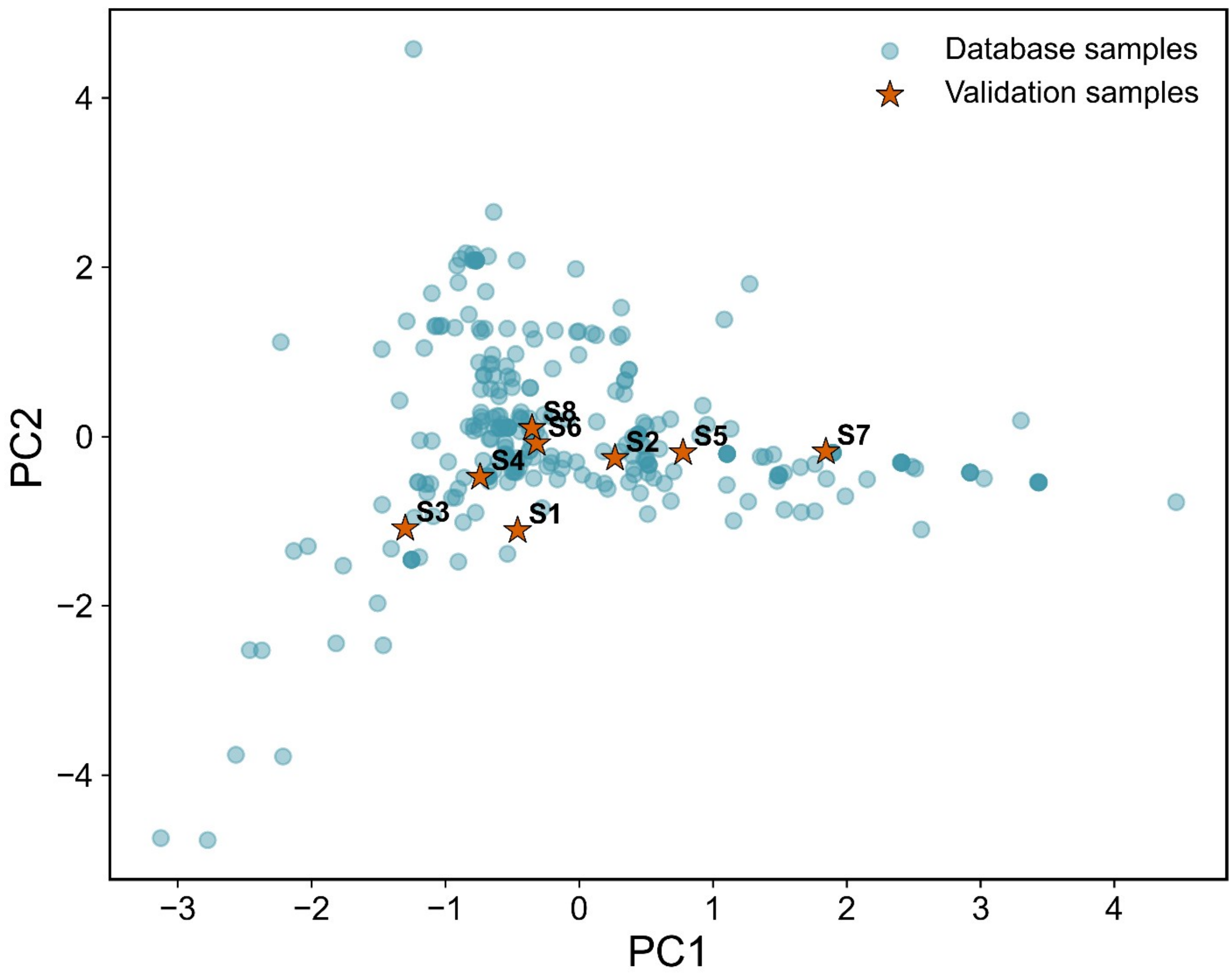

Fig. S7 Principal component analysis (PCA) of the literature-derived synthesis dataset and experimental validation samples. The blue circles represent the synthesis conditions collected from the literature database, while the orange stars denote the eight experimental validation samples.

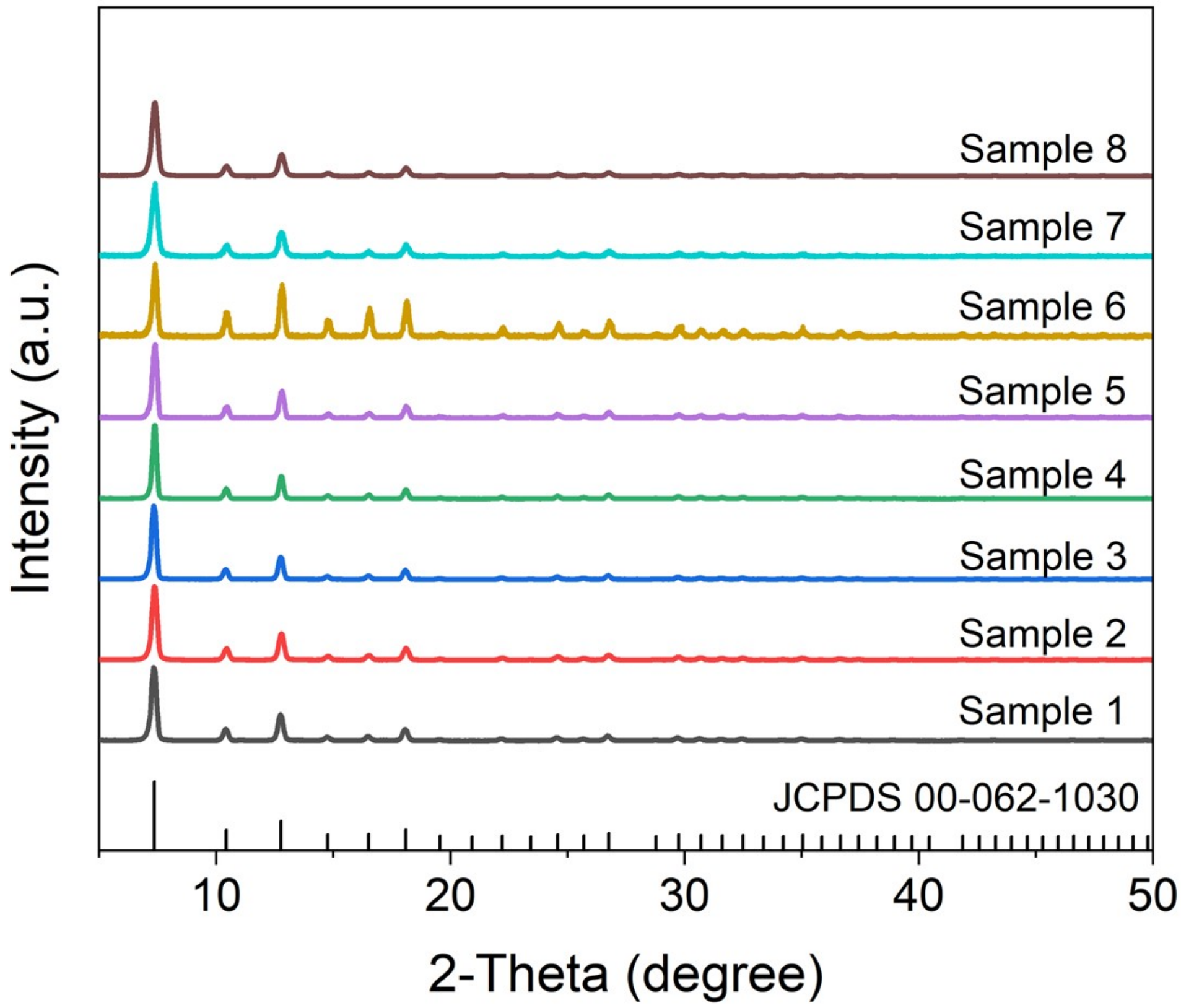

Fig. S8 Normalized XRD patterns of the prepared ZIF-8 samples in comparison with the standard reference pattern (JCPDS 00-062-1030).

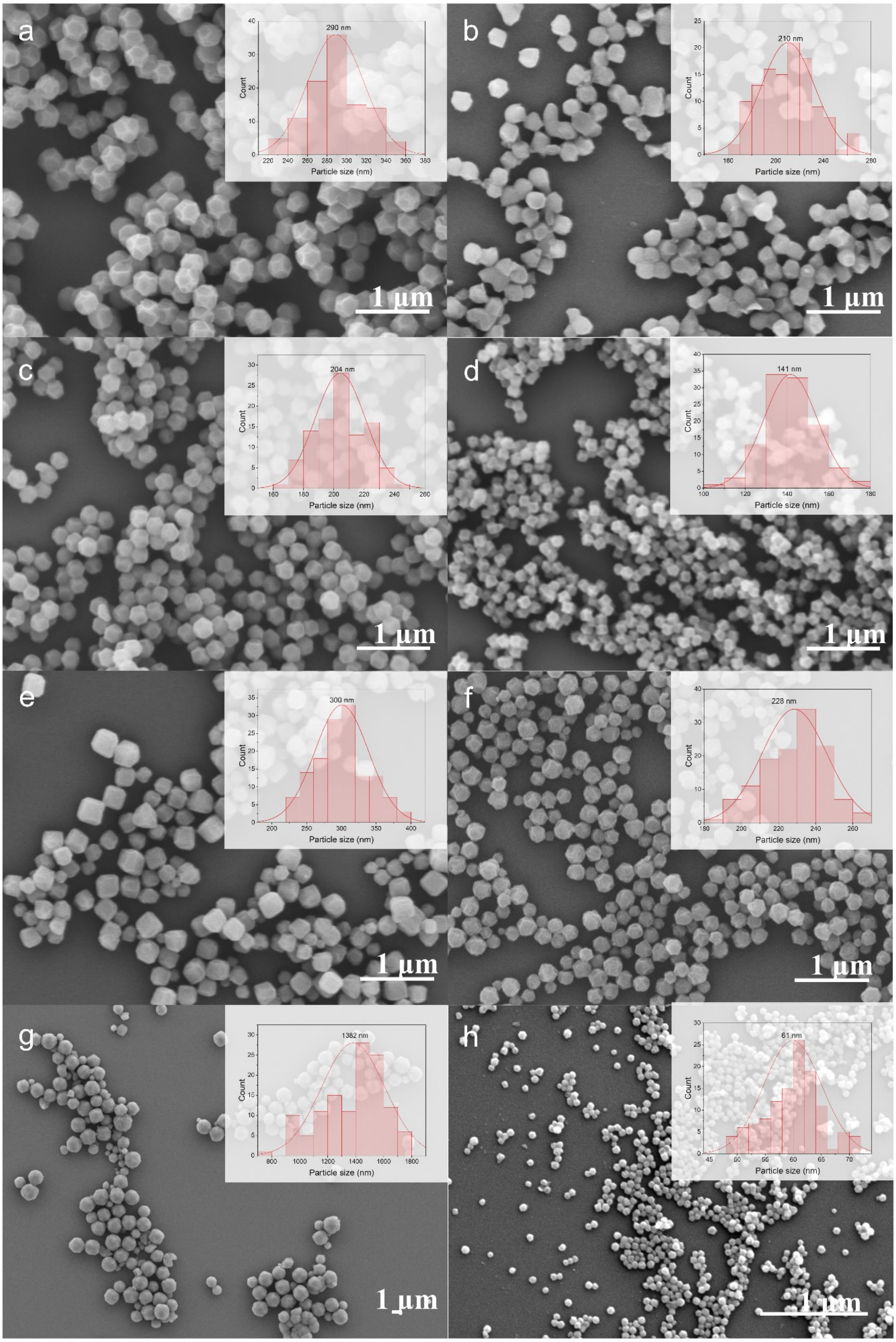

Fig. S9 SEM images of all validation samples. The insets show the corresponding particle-size distributions obtained from statistical analysis of more than 100 particles for each sample.

Reference

1 M. Ahmad, V. Martin-Gil, V. Perfilov, P. Sysel and V. Fila, *Sep. Purif. Technol.*, 2018, **207**, 523–534.

2 M. Ahmadi, E. Asadian, M. Mosayebnia, S. Dadashzadeh, S. Shahhosseini and F. Ghorbani-Bidkorpeh, *Iran. J. Pharm. Res.*, 2024, **23**, 44928.

3 I. Aljundi, *Desalination*, 2017, **420**, 12–20.

4 V. Armel, J. Hannauer and F. Jaouen, *Catalysts*, 2015, **5**, 1333–1351.

5 P. Balkanloo, A. Marjani and M. Mahmoudian, *Chem. Eng. J.*, 2024, **496**, 153835.

6 M. Barjasteh, S. Dehnavi, S. Seyedkhani, S. Rahnamaee and M. Golizadeh, *Int. J. Pharm.*, 2022, **629**, 122339.

7 J. Beh, J. Lim, E. Ng and B. Ooi, *Mater. Chem. Phys.*, 2018, **216**, 393–401.

8 H. Cao, Y. Mao, W. Wang, Y. Jin, Y. Gao, M. Zhang, X. Zhao, J. Sun and Z. Song, *Desalination*, 2023, **550**, 116373.

9 N. Chang, Z. Gu and X. Yan, *J. Am. Chem. Soc.*, 2010, **132**, 13645–13647.

10 K. Chen, P. Wang, A. Gu, E. Miensah, C. Gong, P. Mao, Y. Jiao, K. Chen, Y. Liu and Y. Yang, *Microporous Mesoporous Mater.*, 2022, **339**, 111983.

11 W. Chi, S. Hwang, S. Lee, S. Park, Y. Bae, D. Ryu, J. Kim and J. Kim, *J. Membr. Sci.*, 2015, **495**, 479–488.

12 M. Chin, C. Cisneros, S. Araiza, K. Vargas, K. Ishihara and F. Tian, *RSC Adv.*, 2018, **8**, 26987–26997.

13 K. Cho, H. An, X. Do, K. Choi, H. Yoon, H. Jeong, J. Lee and K. Baek, *J. Mater. Chem. A*, 2018, **6**, 18912–18919.

14 A. Chowdhuri, B. Das, A. Kumar, S. Tripathy, S. Roy and S. Sahu, *Nanotechnology*, 2017, **28**, 095102.

15 J. Cravillon, S. Münzer, S. Lohmeier, A. Feldhoff, K. Huber and M. Wiebcke, *Chem. Mater.*, 2009, **21**, 1410–1412.

16 J. Cravillon, R. Nayuk, S. Springer, A. Feldhoff, K. Huber and M. Wiebcke, *Chem. Mater.*, 2011, **23**, 2130–2141.

17 J. Cravillon, C. Schröder, R. Nayuk, J. Gummel, K. Huber and M. Wiebcke, *Angew. Chem.-Int. Ed.*, 2011, **50**, 8067–8071.

18 A. De, Y. Reddy, S. Paul, V. Sharma, V. Tippavajhala and J. Bhaumik, *Mol. Pharm.*, 2025, **22**, 827–839.

19 N. Demir, B. Topuz, L. Yilmaz and H. Kalipcilar, *Microporous Mesoporous Mater.*, 2014, **198**, 291–300.

20 Y. Ding, Y. Xu, B. Ding, Z. Li, F. Xie, F. Zhang, H. Wang, J. Liu and X. Wang, *Colloids Surf. Physicochem. Eng. Asp.*, 2017, **520**, 661–667.

21 K. Eum, K. Jayachandrababu, F. Rashidi, K. Zhang, J. Leisen, S. Graham, R. Lively, R. Chance, D. Sholl, C. Jones and S. Nair, *J. Am. Chem. Soc.*, 2015, **137**, 4191–4197.

22 X. Fan, J. Zhou, T. Wang, J. Zheng and X. Li, *RSC Adv.*, 2015, **5**, 58595–58599.

23 T. Gao, H. Li, F. Zhou, M. Gao, S. Liang and M. Luo, *Desalination*, 2019, **451**, 133–138.

24 W. He, X. Guo, J. Zheng, J. Xu, T. Hayat, N. Alharbi and M. Zhang, *Inorg. Chem.*, 2019, **58**, 7255–7266.

25 K. Heinz, S. Rogge, A. Kalytta-Mewes, D. Volkmer and H. Bunzen, *Inorg. Chem. Front.*, 2023, **10**, 4763–4772.

26 T. Imae, A. Rahmawati, A. Berhe and M. Kebede, *ACS Appl. Nano Mater.*, 2022, **5**, 16842–16852.

27 S. Jampa, A. Unnarkat, R. Vanshpati, S. Pandian, M. Sinha and S. Dharaskar, *Environ. Technol. Innov.*, 2020, **19**, 100927.

28 A. Jana, A. Vijayalakshmi, S. Raghunathan, A. Shankar, K. Sainath and A. Modi, *Colloids Surf. C Environ. Asp.*, 2024, **2**, 100030.

29 M. Jian, B. Liu, R. Liu, J. Qu, H. Wang and X. Zhang, *RSC Adv.*, 2015, **5**, 48433–48441.

30 J. Jiang, C. Yang and X. Yan, *ACS Appl. Mater. Interfaces*, 2013, **5**, 9837–9842.

31 Y. Jiang, H. Liu, X. Tan, L. Guo, J. Zhang, S. Liu, Y. Guo, J. Zhang, H. Wang and W. Chu, *ACS Appl. Mater. Interfaces*, 2017, **9**, 25239–25249.

32 X. Jiang, S. He, G. Han, J. Long, S. Li, C. Lau, S. Zhang and L. Shao, *ACS Appl. Mater. INTERFACES*, 2021, **13**, 11296–11305.

33 A. Jomekian, R. Behbahani, T. Mohammadi and A. Kargari, *J. Nat. Gas Sci. Eng.*, 2016, **31**, 562–574.

34 A. Jomekian, B. Bazooyar, R. Behbahani, T. Mohammadi and A. Kargari, *J. Membr. Sci.*, 2017, **524**, 652–662.

35 A. Karimi, A. Khataee, V. Vatanpour and M. Safarpour, *Sep. Purif. Technol.*, 2019, **229**, 115838.

36 S. Kawada, T. Otsubo, T. Horie, Y. Komoda, N. Ohmura, H. Asano, R. Hidema, H. Suzuki, K. Taniya, Y. Ichihashi and S. Nishiyama, *CrystEngComm*, 2022, **24**, 7378–7386.

37 I. Khay, G. Chaplais, H. Nouali, C. Marichal and J. Patarin, *RSC Adv.*, 2015, **5**, 31514–31518.

38 K. Kida, M. Okita, K. Fujita, S. Tanaka and Y. Miyake, *CrystEngComm*, 2013, **15**, 1794–1801.

39 J. Kim, S. Moon, H. Wang, S. Kim and Y. Lee, *J. Membr. Sci.*, 2019, **582**, 381–390.

40 S. Kim, J. Lee, J. Bae and J. Lee, *Nanomaterials*, 2024, **14**, 284.

41 O. Kolmykov, N. Chebbat, J. Commenge, G. Medjandi and R. Schneider, *Tetrahedron Lett.*, 2016, **57**, 5885–5888.

42 W. Kong, M. Serdechnova, V. Kasneryk, D. Gao, H. Wang, X. Xie, C. Blawert, M. Zheludkevich and Y. Zhang, *Surf. Coat. Technol.*, 2024, **483**, 130812.

43 S. Kulkarni, A. Pandey, A. Nikam, S. Nannuri, S. George, S. Fayaz, A. Vincent and S. Mutalik, *Int. J. Biol. Macromol.*, 2021, **178**, 444–463.

44 G. Kumari, K. Jayaramulu, T. Maji and C. Narayana, *J. Phys. Chem. A*, 2013, **117**, 11006–11012.

45 L. Lai, Y. Yeong, N. Ani, K. Lau and A. Shariff, *Part. Sci. Technol.*, 2014, **32**, 520–528.

46 Q. Lai, Y. Zhao, Y. Liang, J. He and J. Chen, *Adv. Funct. Mater.*, 2016, **26**, 8334–8344.

47 Q. Lai, L. Zheng, Y. Liang, J. He, J. Zhao and J. Chen, *ACS Catal.*, 2017, **7**, 1655–1663.

48 T. Lee, H. Kim, W. Cho, D. Han, M. Ridwan, C. Yoon, J. Lee, N. Choi, K. Ha, A. Yip and J. Choi, *J. Phys. Chem. C*, 2015, **119**, 8226–8237.

49 J. Lee, D. Kim, H. Shin, S. Yoo, H. Kwon and J. Kim, *J. Ind. Eng. Chem.*, 2019, **72**, 374–379.

50 T. Lee, J. Oh, S. Hong, J. Lee, S. Roh, S. Kim and H. Park, *J. Membr. Sci.*, 2019, **570**, 23–33.

51 J. Li, Y. Wu, Z. Li, B. Zhang, M. Zhu, X. Hu, Y. Zhang and F. Li, *J. Phys. Chem. C*, 2014, **118**, 27382–27387.

52 H. Li, D. Fu, X. Zhang, G. Han and F. Zhang, *ChemistrySelect*, 2017, **2**, 7530–7534.

53 X. Li, C. Hao, B. Tang, Y. Wang, M. Liu, Y. Wang, Y. Zhu, C. Lu and Z. Tang, *Nanoscale*, 2017, **9**, 2178–2187.

54 Y. Li, J. Kim, J. Wang, N. Liu, Y. Bando, A. Alshehri, Y. Yarnauchi, C. Hou and K. Wu, *Nanoscale*, 2018, **10**, 14852–14859.

55 P. Li, J. Li, X. Feng, J. Li, Y. Hao, J. Zhang, H. Wang, A. Yin, J. Zhou, X. Ma and B. Wang, *Nat. Commun.*, 2019, **10**, 2177.

56 Y. Li, J. Henzie, T. Park, J. Wang, C. Young, H. Xie, J. Yi, J. Li, M. Kim, J. Kim, Y. Yamauchi and J. Na, *Bull. Chem. Soc. Jpn.*, 2020, **93**, 176–181.

57 N. Li, Z. Wang, M. Wang, M. Gao, H. Wu, S. Zhao and J. Wang, *J. Membr. Sci.*, 2021, **624**, 119095.

58 Y. Li, Z. Lin, X. Wang, Z. Duan, P. Lu, S. Li, D. Ji, Z. Wang, G. Li, D. Yu and W. Liu, *Sep. Purif. Technol.*, 2021, **270**, 118794.

59 T. Li, Y. Wang, X. Wang, C. Cheng, K. Zhang, J. Yang, G. Han, Z. Wang, X. Wang and L. Wang, *Membranes*, 2022, **12**, 122.

60 J. Li, Y. Hua, Y. Gao, S. Li, T. Kedzierski, E. Mijowska, P. Chu, R. Holze, Y. He, W. Bi and X. Chen, *SMALL*, 2024, **20**, 2406187.

61 O. Linder-Patton, T. de Prinse, S. Furukawa, S. Bell, K. Sumida, C. Doonan and C. Sumby, *CrystEngComm*, 2018, **20**, 4926–4934.

62 X. Liu, Y. Li, Y. Ban, Y. Peng, H. Jin, H. Bux, L. Xu, J. Caro and W. Yang, *Chem. Commun.*, 2013, **49**, 9140–9142.

63 Q. Liu, Z. Low, L. Li, A. Razmjou, K. Wang, J. Yao and H. Wang, *J. Mater. Chem. A*, 2013, **1**, 11563–11569.

64 D. Liu, X. Ma, H. Xi and Y. Lin, *J. Membr. Sci.*, 2014, **451**, 85–93.

65 G. Liu, Y. Xu, Y. Han, J. Wu, J. Xu, H. Meng and X. Zhang, *Dalton Trans.*, 2017, **46**, 2114–2121.

66 Y. Luo, S. Fan, W. Yu, Z. Wu, D. Cullen, C. Liang, J. Shi and C. Su, *Adv. Mater.*, 2018, **30**, 1704576.

67 C. Luo, F. Fu, X. Yang, J. Wei, C. Wang, J. Zhu, D. Huang, D. Astruc and P. Zhao, *ChemCatChem*, 2019, **11**, 1643–1649.

68 J. McEwen, J. Hayman and A. Yazaydin, *Chem. Phys.*, 2013, **412**, 72–76.

69 A. Mittal, S. Gandhi and I. Roy, *Sci. Rep.*, 2022, **12**, 10331.

70 A. Mohammadi, E. Jafarpour, K. Mirzaei, A. Shojaei, P. Jafarpour, M. Eyni, S. Mirzaei and H. Molavi, *ACS Appl. Mater. Interfaces*, 2024, **16**, 3862–3875.

71 D. Muñoz-Gil and F. Figueiredo, *Nanomaterials*, 2019, **9**, 1369.

72 Q. Nguyen, K. Jeong, Y. Lee and K. Baek, *J. COsub2sub Util.*, 2023, **70**, 102451.

73 M. Nie, S. Lu, D. Lei, C. Yang and Z. Zhao, *J. Electrochem. Soc.*, 2017, **164**, H952–H957.

74 Y. Pan, Y. Liu, G. Zeng, L. Zhao and Z. Lai, *Chem. Commun.*, 2011, **47**, 2071–2073.

75 S. Pandey, B. Sharmah, P. Manna, Z. Chawngthu, S. Kumar, A. Trivedi, S. Saha and J. Das, *J. Mol. Struct.*, 2024, **1312**, 138452.

76 J. Patterson, P. Abellan, M. Denny, C. Park, N. Browning, S. Cohen, J. Evans and N. Gianneschi, *J. Am. Chem. Soc.*, 2015, **137**, 7322–7328.

77 S. Qin, X. Du, K. Wang, D. Wang, J. Zheng, H. Xu, X. Wei and Y. Yuan, *Int. J. Pharm.*, 2023, **642**, 123167.

78 J. Qiu, X. Xu, B. Liu, Y. Guo, H. Wang, L. Yu, Y. Jiang, C. Huang, B. Fan, Z. Zeng and L. Li, *ChemistrySelect*, 2022, **7**, 202203273.

79 S. Saghir and Z. Xiao, *Mater. Res. Bull.*, 2021, **141**, 111372.

80 A. Schejn, L. Balan, V. Falk, L. Aranda, G. Medjahdi and R. Schneider, *CrystEngComm*, 2014, **16**, 4493–4500.

81 P. Schneider, K. Kollmannsberger, C. Cesari, R. Khare, M. Boniface, B. Cuenya, T. Lunkenbein, M. Elsner, S. Zacchini, A. Bandarenka, J. Warnan and R. Fischer, *Chemelectrochem*, 2024, **11**, 202300476.

82 H. Seo, J. Kang, H. Kim, S. Jang, J. Kim, S. Choi, H. Eom, O. Kwon, J. Shin, J. Park, D. Yoo, S. Jeong, S. Noh, C. Park, M. Seol, S. Park and I. Nam, *Korean J. Chem. Eng.*, 2025, **42**, 1529–1538.

83 H. Seo, Y. Lee, H. Kim, S. Jang, J. Kim, J. Kang, H. Eom, O. Kwon, J. Shin, J. Park, S. Choi, Y. Bae, C. Park, M. Seol, H. Song, S. Park and I. Nam, *J. Alloys Compd.*, 2025, **1013**, 178578.

84 S. Sharma, P. Utpalla, J. Bahadur, A. Das, J. Prakash and P. Pujari, *J. Phys. Chem. C*, 2020, **124**, 25291–25298.

85 H. Shen, H. Zhao, E. Benassi, L. Chou and H. Song, *CrystEngComm*, 2023, **25**, 3308–3316.

86 J. Shi, X. Wang, S. Zhang, L. Tang and Z. Jiang, *J. Mater. Chem. B*, 2016, **4**, 2654–2661.

87 Y. Si, X. Li, G. Yang, X. Mie and L. Ge, *J. Mater. Sci.*, 2020, **55**, 13049–13061.

88 Y. Song, S. Han, S. Liu, R. Sun, L. Zhao and C. Yan, *ACS Appl. Mater. Interfaces*, 2023, **15**, 25339–25353.

89 J. Sánchez-Laínez, B. Zornoza, S. Friebe, J. Caro, S. Cao, A. Sabetghadam, B. Seoane, J. Gascon, F. Kapteijn, C. Le Guillouzer, G. Clet, M. Daturi, C. Téllez and J. Coronas, *J. Membr. Sci.*, 2016, **515**, 45–53.

90 D. Ta, H. Nguyen, B. Trinh, Q. Le, H. Ta and H. Nguyen, *Can. J. Chem. Eng.*, 2018, **96**, 1518–1531.

91 S. Tanaka, K. Kida, M. Okita, Y. Ito and Y. Miyake, *Chem. Lett.*, 2012, **41**, 1337–1339.

92 S. Tanaka, K. Fujita, Y. Miyake, M. Miyamoto, Y. Hasegawa, T. Makino, S. Van der Perre, J. Saint Remi, T. Van Assche, G. Baron and J. Denayer, *J. Phys. Chem. C*, 2015, **119**, 28430–28439.

93 A. Thomas, P. Immanuel, N. Prasad, A. Goldreich, J. Prilusky, R. Carmieli and L. Yadgarov, *Nanoscale Adv.*, 2025, **7**, 3764–3777.

94 T. Tian, M. Wharmby, J. Parra, C. Ania and D. Fairen-Jimenez, *Dalton Trans.*, 2016, **45**, 6893–6900.

95 N. Torad, M. Hu, Y. Kamachi, K. Takai, M. Imura, M. Naito and Y. Yamauchi, *Chem. Commun.*, 2013, **49**, 2521–2523.

96 N. Tran, J. Ki and M. Othman, *Sep. Purif. Technol.*, 2020, **233**, 116026.

97 C. Tsai and E. Langner, *Microporous Mesoporous Mater.*, 2016, **221**, 8–13.

98 S. Van Cleuvenbergen, I. Stassen, E. Gobechiya, Y. Zhang, K. Markey, D. De Vos, C. Kirschhock, B. Champagne, T. Verbiest and M. van der Veen, *Chem. Mater.*, 2016, **28**, 3203–3209.

99 S. Venna, J. Jasinski and M. Carreon, *J. Am. Chem. Soc.*, 2010, **132**, 18030–18033.

100 Z. Wang, T. Yan, J. Fang, L. Shi and D. Zhang, *J. Mater. Chem. A*, 2016, **4**, 10858–10868.

101 H. Wang, Y. Wang, A. Jia, C. Wang, L. Wu, Y. Yang and Y. Wang, *Catal. Sci. Technol.*, 2017, **7**, 5572–5584.

102 Z. Wang, H. Jin, T. Meng, K. Liao, W. Meng, J. Yang, D. He, Y. Xiong and S. Mu, *Adv. Funct. Mater.*, 2018, **28**, 1802596.

103 J. Wang, G. Han, L. Wang, L. Du, G. Chen, Y. Gao, Y. Ma, C. Du, X. Cheng, P. Zuo and G. Yin, *Small*, 2018, **14**, 1704282.

104 F. Wang, T. Zheng, R. Xiong, P. Wang and J. Ma, *Chemosphere*, 2019, **233**, 524–531.

105 J. Wang, X. Zhao, H. Qu, J. Xu and J. Ma, *Appl. Surf. Sci.*, 2023, **615**, 156181.

106 M. Weber, T. Baker, B. Dao, C. Kwon and F. Tian, *Cryst. Growth Des.*, 2020, **20**, 2305–2312.

107 Y. Xue, Y. Liu, C. Wang and Y. Yao, *J. Inorg. Organomet. Polym. Mater.*, 2024, **34**, 6159–6167.

108 M. Yahia, Q. Le, N. Ismail, M. Essalhi, O. Sundman, A. Rahimpour, M. Dal-Cin and N. Tavajohi, *Microporous Mesoporous Mater.*, 2021, **312**, 110761.

109 R. Yang, X. Yan, Y. Li, X. Zhang and J. Chen, *ACS Appl. Mater. Interfaces*, 2017, **9**, 42482–42491.

110 H. Yang, X. Guo, R. Chen, Q. Liu, J. Liu, J. Yu, C. Lin, J. Wang and M. Zhang, *Eur. Polym. J.*, 2021, **144**, 110212.

111 H. Yin, H. Kim, J. Choi and A. Yip, *Chem. Eng. J.*, 2015, **278**, 293–300.

112 C. Zhang, R. Lively, K. Zhang, J. Johnson, O. Karvan and W. Koros, *J. Phys. Chem. Lett.*, 2012, **3**, 2130–2134.

113 K. Zhang, R. Lively, C. Zhang, R. Chance, W. Koros, D. Sholl and S. Nair, *J. Phys. Chem. Lett.*, 2013, **4**, 3618–3622.

114 L. Zhang, Z. Su, F. Jiang, L. Yang, J. Qian, Y. Zhou, W. Li and M. Hong, *Nanoscale*, 2014, **6**, 6590–6602.

115 C. Zhang, J. Gee, D. Sholl and R. Lively, *J. Phys. Chem. C*, 2014, **118**, 20727–20733.

116 W. Zhang, Z. Wu, H. Jiang and S. Yu, *J. Am. Chem. Soc.*, 2014, **136**, 14385–14388.

117 H. Zhang, W. Jiang, R. Liu, J. Zhang, D. Zhang, Z. Li and Y. Luan, *ACS Appl. Mater. Interfaces*, 2017, **9**, 19687–19697.

118 M. Zhang, X. Shi, X. Dai, C. Huo, J. Xie, X. Li and X. Wang, *J. Mater. Sci.*, 2018, **53**, 7083–7093.

119 H. Zhang, M. Zhao and Y. Lin, *Microporous Mesoporous Mater.*, 2019, **279**, 201–210.

120 H. Zhang, M. Zhao, Y. Yang and Y. Lin, *Microporous Mesoporous Mater.*, 2019, **288**, 109568.

121 Q. Zhang, C. Tang, M. Han, Z. Tong, L. Cao, S. Dong, Y. Yang, J. Li, X. Cao, J. Zhang, K. Wang and S. Zhang, *ACS Appl. Polym. Mater.*, 2025, **7**, 7113–7121.

122 Y. Zhao, Y. Pan, W. Liu and L. Zhang, *Chem. Lett.*, 2015, **44**, 758–760.

123 H. Zheng, Y. Zhang, L. Liu, W. Wan, P. Guo, A. Nyström and X. Zou, *J. Am. Chem. Soc.*, 2016, **138**, 962–968.

124 J. Zhou, X. Yu, X. Fan, X. Wang, H. Li, Y. Zhang, W. Li, J. Zheng, B. Wang and X. Li, *J. Mater. Chem. A*, 2015, **3**, 8272–8275.

125 L. Zhou, H. Li, D. Wang, W. Jiang, Y. Wu, L. Shang, C. Guo, C. Liu and B. Ren, *Electroanalysis*, 2023, **35**, 291–301.
126 M. Zhu, S. Venna, J. Jasinski and M. Carreon, *Chem. Mater.*, 2011, **23**, 3590–3592.
127 M. Zhu, D. Srinivas, S. Bhogeswararao, P. Ratnasamy and M. Carreon, *Catal. Commun.*, 2013, **32**, 36–40.
128 Y. Zhu, J. Ciston, B. Zheng, X. Miao, C. Czarnik, Y. Pan, R. Sougrat, Z. Lai, C. Hsiung, K. Yao, I. Pinnau, M. Pan and Y. Han, *Nat. Mater.*, 2017, **16**, 532–536.
129 Q. Zhu, W. Zhuang, Y. Chen, Z. Wang, B. Hernandez, J. Wu, P. Yang, D. Liu, C. Zhu, H. Ying and Z. Zhu, *ACS Appl. Mater. Interfaces*, 2018, **10**, 16066–16076.
130 R. Zhu, L. Wang, H. Zhang, C. Liu and Z. Wang, *Sep. Purif. Technol.*, 2024, **335**, 126209.
131 N. J. van Eck and L. Waltman, *Scientometrics*, 2010, **84**, 523–538.
